# Multiple programmed cell death patterns predict the prognosis and drug sensitivity in gastric cancer

**DOI:** 10.3389/fimmu.2025.1511453

**Published:** 2025-02-04

**Authors:** Qiying Song, Shihe Liu, Di Wu, Aizhen Cai

**Affiliations:** Department of General Surgery, The First Medical Center of Chinese People's Liberation Army General Hospital, Beijing, China

**Keywords:** gastric cancer, program cell death, prognosis, immunotherapy, drug sensitivity

## Abstract

**Background:**

Gastric cancer (GC) is a malignant tumor with poor prognosis. The diverse patterns of programmed cell death (PCD) are significantly associated with the pathogenesis and progression of GC, and it has the potential to serve as prognostic and drug sensitivity indicators for GC.

**Method:**

The sequencing data and clinical characteristics of GC patients were downloaded from The Cancer Genome Atlas and GEO databases. LASSO cox regression method was used to screen feature genes and develop the PCD score (PCDS). Immune cell infiltration, immune checkpoint expression, Tumor Immune Dysfunction and Exclusion (TIDE) algorithm and drug sensitivity analysis were used to explore immunotherapy response. By integrating PCDS with clinical characteristics, we constructed and validated a nomogram that demonstrated robust predictive performance.

**Results:**

We screened nine PCD-related genes (SERPINE1, PLPPR4, CDO1, MID2, NOX4, DYNC1I1, PDK4, MYB, TUBB2A) to create the PCDS. We found that GC patients with high PCDS experienced significantly poorer prognoses, and PCDS was identified as an independent prognostic factor. Furthermore, there was a significant difference in immune profile between high PCDS and low PCDS groups. Additionally, drug sensitivity analysis indicated that patients with a high PCDS may exhibit resistance to immunotherapy and standard adjuvant chemotherapy regimens; however, they may benefit from the FDA-approved drug Dasatinib.

**Conclusion:**

Overall, we confirmed that the PCDS is a prognostic risk factor and a valuable predictor of immunotherapy response in GC patients, which provides new evidence for the potential application of GC.

## Introduction

Gastric cancer (GC) represents the fifth most prevalent malignancy globally, with its mortality rate ranking fourth among all malignant tumors worldwide (1). In recent years, the ongoing advancements in tumor immunotherapy, epitomized by inhibitors targeting programmed death receptor 1 (PD-1) and its ligand PD-L1, have demonstrated remarkable efficacy across various solid tumors, including gastric cancer (2, 3). However, the overall effective rate of PD-1/PD-L1 inhibitors in the unselected population of solid tumors is less than 20% (2, 3). Consequently, an imperative need arises to devise precise and robust models to identify gastric cancer patients who are susceptible to tumor immunotherapy, thereby enabling individualized clinical interventions.

Programmed cell death (PCD), also known as regulated cell death, refers to the self-regulating process in which cells die under the control of specific genes and the precise coordination of various mechanisms. The main goal of PCD is to maintain the stability of the internal cellular environment. According to triggering stress, morphological characteristics, regulatory signaling pathways and effector molecules, PCD can be divided into apoptosis, necroptosis, pyroptosis, ferroptosis, entotic cell death (entosis), netotic cell death (NETosis), parthanatos, lysosome-dependent cell death (LDCD), autophagy-dependent cell death (ADCD), alkaliptosis, oxeiptosis, cuproptosis and paraptosis, immunogenic cell death (ICD) (4, 5). The activation of apoptosis mainly includes the extrinsic and the intrinsic pathway (5). Extrinsic apoptosis is mediated by the activation of plasma membrane-localized death receptors (such as TNFR1, Fas) by their cognate ligands (such as TNF, FasL) (6). Intrinsic apoptosis can be activated by BCL-2 family proteins induced mitochondrial outer membrane permeabilization by releasing cytochrome c and mediated by caspase-3/7/9 (6). Necroptosis is a form of death triggered by extracellular stimuli activating death receptors, which causes phosphorylation of receptor-interacting protein kinase (RIPK), leading to the recruitment of mixed lineage kinase domain-like (MLKL), necroptosis mainly depends on the activation of RIPK1 and RIPK3 (7). Pyroptosis is triggered by caspase-1-driven cleavage of the pore-forming protein gasdermin D (GSDMD), then the N-terminal fragment of GSDME will lead to the formation of cell membrane pores and thereby induce pyroptosis (8). Ferroptosis is an iron-dependent form of cell death characterized by the accumulation of lipid peroxides (9). The process includes iron accumulation, reactive oxygen species (ROS) activation, reduced cysteine uptake, depletion of glutathione (GSH), and activation of the mitogen-activated protein kinase (MAPK) system (10). Cuproptosis is a newly discovered type. It is related to the imbalance of intracellular copper metabolism. Excessive copper directly binds to lipoylated proteins in the tricarboxylic acid (TCA) cycle of mitochondria, leading to the abnormal aggregation of lipoylated proteins and the loss of iron-sulfur cluster proteins in respiratory chain complexes, causing a protein toxic stress response and ultimately leading to cell death (11). Entosis is triggered by autophagosomes formed by the cell membrane engulfing its cytoplasmic proteins or organelles. Its regulation mainly depends on the mTOR pathway, including signal pathways such as PI3K-AKT-mTOR and AMPK-TSC1/2-mTOR (12). LDCD is mainly achieved through changes in lysosomal membrane permeability (LMP). When LMP increases, the release of cathepsin B and cathepsin D in lysosomes will trigger lysosome-dependent cell death (6). NETosis is a form of regulated cell death (RCD) driven by neutrophil extracellular trap (NET), which is regulated by NADPH oxidase-mediated ROS production and histone citrullination (13). Parthanatos is mediated by poly (ADP-ribose) polymerase 1 (PARP1), which is caused by DNA damage. PARP1 hyper-activation stimulates apoptosis-inducing factor (AIF) nucleus translocation, and accelerates nicotinamide adenine dinucleotide (NAD+) and adenosine triphosphate (ATP) depletion, leading to DNA fragmentation (14). Alkaliptosis is a pH-dependent cell death process triggered by the small molecular compound JTC801 (15). ADCD, a phagocytic biological process, can disintegrate damaging proteins or organelles through lysosomal fusion (16). Oxeiptosis is activated in response to oxidative stress induced by ROS or ROS-generating agents and characterized by the activation of the KEAP1/PGAM5/AIFM1 signaling pathway (4). ICD is triggered by the release of damage-associated molecular patterns (DAMPs) from dying cells, which can trigger an adaptive immune response, release antigens, reverse the tumor immunosuppressive microenvironment, and improve the sensitivity of immunotherapy (17). Paraptosis is characterized by the swelling and vacuolization of the endoplasmic reticulum (ER) and mitochondria, resulting in the formation of large cytoplasmic vacuoles (4).

Increasing evidence shows that PCD plays a critical role in cancer initiation and progression (18–23). Cancer cell death has been confirmed as fundamental in the remodeling of the tumor immune microenvironment (TIME) (24). For instance, tumor cell fragments serve as antigens, which are captured, processed, and presented by conventional dendritic cells (cDCs) (25). Certain types of cell death, such as necroptosis and pyroptosis, release DAMPs and inflammatory cytokines due to cell membrane rupture (26). Conversely, some studies suggested that cell death can also directly or indirectly cause immunosuppression by recruiting myeloid cells (such as immunosuppressive macrophage subsets) (27). Considering the inherent connection between the TIME and the efficacy of immunotherapy, the recognition and induction of PCD forms to potentiate the immune response against cancer, particularly in the context of immune checkpoint inhibitors (ICIs), is of paramount importance (26). Furthermore, mounting evidence indicates that cancer patients with varying prognoses often exhibit distinct differences in TIME and their response to ICIs (28, 29).

In the past few years, many scholars have developed prediction models with characteristic genes of a single form of PCD, and have achieved moderate prediction accuracy in predicting cancer prognosis and drug resistance (30). However, interactions have been identified within signaling pathways that regulate different forms of cell death (31). While each form of cell death has its mechanism, they are not independent individuals and still have connections with each other (31). For instance, reactive oxygen species (ROS) is an indispensable component in the process of ferroptosis and can also participate in apoptosis, autophagy, necroptosis, and pyroptosis (32, 33). Lysosomal membrane permeabilization (LMP) not only participates in lysosome-dependent cell death but also amplifies cell death signals and increases the complexity of cell death under the induction of autophagy, necroptosis, and ferroptosis (16).

Therefore, a prediction model incorporating multiple forms of PCD may provide a more comprehensive representation of tumor characteristics compared to a model focusing on a single type. In this study, we collected 14 PCD pattern-related genes to identify biomarkers and establish a PCD score (PCDS) signature, aiming to predict the TIME, prognosis, and responsiveness to immunotherapy in GC. In the future, this may assist doctors in making individualized clinical treatment.

## Materials and methods

### Data collection

The genes associated with PCD were sourced from Molecular Signature Database (MSigDB), Human Gene Database (GeneCards), Kyoto encyclopedia of genes and genomes (KEGG), as well as review articles (4, 34, 35). Ultimately, 14 PCD patterns-related genes were assembled, including apoptosis (n = 860), necroptosis (203), pyroptosis (n = 71), ferroptosis (n = 591), cuproptosis (n = 73), entotic cell death (n = 39), NETosis (n = 85), parthanatos (n = 23), lysosome-dependent Cell Death (n = 220), autophagy-dependent cell death (n = 735), alkaliptosis (n = 7), oxeiptosis (n = 5), paraptosis (n = 7), immunogenic cell death (ICD) (n = 34). The genes of different PCD patterns overlap to some extent. Eventually, 2250 different genes were included in this study (See **Supplementary Table 1**).

For the training dataset, transcriptomic profiles along with corresponding clinical data were obtained for 412 GC patients and 36 control subjects from The Cancer Genome Atlas (TCGA) STAD database. For the validation cohorts, 433 GC patients in the GSE8443 and 357 GC patients in the GSE84433 which were generated on the GPL960 platform in the Gene Expression Omnibus (GEO) were retrieved.

### Identification and enrichment analysis of differentially expressed genes

The original transcriptome count data of 412 GC patients from TCGA-GC and 36 normal tissues in the TCGA cohort were compared. Then, the “limma”, “DEseq2”, and “edgR” packages were used to screen out differentially expressed genes (DEGs) related to PCD with the screening criteria were FDR < 0.05 and |log2FC| ≥ 1 (36). Considering the analysis error caused by using any of “Deseq2”, “limma”, or “edgR” separately, the intersection of their outputs utilized for subsequent analyses (37). And the “clusterProfiler” package in R software was used to evaluate possible biological pathways of PCD related DEGs (38). To investigate the somatic mutation data within GC patients, the “maftools” package was applied (39). Copy number variation (CNV) of PCD-related genes was assessed using GISTICS 2.0, with values above 0.2 considered as “gain” and values below -0.2 considered as “losses”. The different characteristics of PCD-related genes were shown in the circus diagram.

### Construction and validation of the multi-gene PCDS signature

385 GC patients in TCGA cohorts with survival data were used for further analysis. A univariate Cox regression analysis was performed to select genes with potentially significant prognostic value (P<0.05). Subsequently, the least absolute shrinkage and selection operator (LASSO) Cox regression method was applied to determine the candidate genes for constructing the optimal signature utilizing the “glmnet” package. Finally, the PCDS for each patient was then calculated using the following formula: PCDS=∑βiGenei. βi represents the risk coefficients, and Genei denotes the expression of each gene. Based on the median PCDS as cutoff value, we divided patients into low and high PCDS groups. Kaplan Meier analysis was used to investigate the relationship between overall survival (OS) and PCDS using “survival” and “survminer” packages. Finally, the GSE84437 cohort with 357 GC patients and GSE84433 cohort with 433 GC patients were served as external validation cohorts to substantiate the predictive capability of the PCDS. The clinical information of three cohorts were presented in **Supplementary Table 2**.

### Pathways and function enrichment analysis

The “clusterProfiler” R package was used to perform gene set enrichment analysis (GSEA) (c2.cp.kegg_legacy.v2023.2.Hs.symbols.gmt) based on transcriptomic data. As indicated in the official literature of GSEA, results with P<0.05 and FDR<0.25 are considered statistically significant (40). Based on somatic mutation data, tumor mutation burden (TMB) between the high and low PCDS groups were compared using the “maftools” package.

### Tumor immune microenvironment analysis and prediction of immunotherapy response

We analyzed the correlation between PCDS and immune modulators. The CIBERSORT, MCPcounter, QUANTISEQ, XCELL, CIBERSORT-ABS, TIMER, and EPIC algorithms were used to analyze the immunological characteristics of the two groups (TIMER, http://timer.cistrome.org/). Furthermore, stromal score, immune score, tumor purity, and ESTIMATE score were calculated using the ESTIMATE algorithm (41). As for the prediction of drug sensitivity response, we estimated the half maximal inhibitory concentration (IC50) values based on drug sensitivity data of GC obtained from Genomics of Drug Sensitivity in Cancer (GDSC). Drug sensitivity was predicted by the “oncoPredict” package (42). Additionally, the prediction of immune therapy response between PCDS groups was performed using the tumor immune dysfunction and exclusion (TIDE) algorithm (http://tide.dfci.harvard.edu/) (43). single-cell RNA sequencing (scRNA-seq) data was collected from GSE183904, which included 25 GC samples and 10 normal gastric tissue samples, and analyzed with “Seurat V4” R package. Risk score was computed by the average risk score of all cells in the sample, and then divided into risk groups by median.

### Establishment and application of prognostic characteristics for PCDS signature

Incorporating clinical characteristics, such as age, gender, and the T, N, and M stages, alongside PCDS, an innovative prognostic nomogram was formulated using multivariate Cox and stepwise regression analyses. Calibration plots were employed to assess the model’s efficacy. Additionally, Receiver Operating Characteristic (ROC) analysis was performed utilizing the “timeROC” package.

### Immunohistochemical analysis

IHC analysis uses the principle of specific antigen-antibody binding to detect and locate target antigens in cells and tissues, mainly with light microscopy. Human Protein Atlas (HPA) database (http://www.proteinatlas.org/) was utilized to implement IHC analysis for key gene expression in GC and normal gastric tissues (44).

### Statistical analysis

Version 4.3.0 of the R software was used for conducting all statistical analyses. Student t-test or Wilcoxon test was used to analyze the differences between the two groups. Kaplan-Meier curves with log-rank tests were used to evaluate the survival. A two-side significance level of P<0.05 is considered significant.

## Results

Firstly, characteristic gene sets of 14 forms of PCD including apoptosis, necroptosis, pyroptosis, ferroptosis, cuproptosis, entosis, NETosis, parthanatos, LDCD, ADCD, alkaliptosis, oxeiptosis, paraptosis, and ICD were collected. The TCGA-STAD database was selected as the training cohort, while the GSE84437 and GSE84433 databases served as the validation cohorts for the prognostic prediction model.

**Figure 1:**
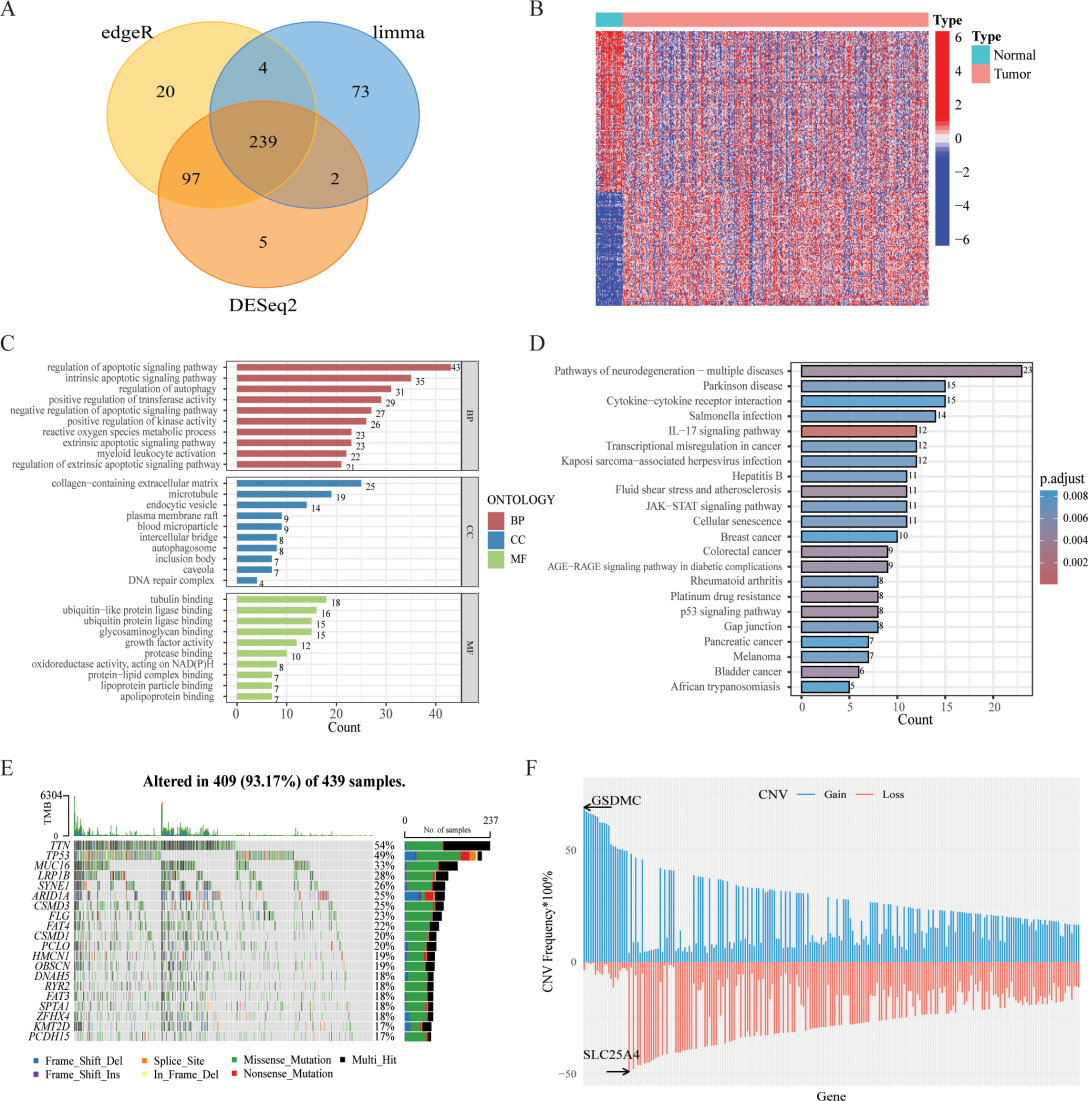
Variant landscape of PCD-related DEGs in GC patients. **(A)** Venn diagram representing PCD-related DEGs between GC and normal tissues. **(B)** Heatmap of the PCD-related DEGs between GC and normal tissues. **(C)** GO enrichment analyses based on the PCD-related DEGs. **(D)** KEGG enrichment analyses based on the PCD-related DEGs. **(E)** An oncoplot of PCD-related DEGs in the TCGA cohort. **(F)** CNV values of PCD-related DEGs in the TCGA cohort.

Integration of the results generated from these three methods identified 239 DEGs (FDR q value < 0.05, |log2FC| > 1), comprising 97 upregulated and 142 downregulated genes between tumor and normal samples (**Figure 1A**, **Supplementary Table 3**). The heatmap of DEGs was shown in **Figure 1B**. Besides, Kyoto Encyclopedia of Genes and Genomes (KEGG) and Gene Ontology (GO) enrichment analysis revealed that these PCD-related DEGs were involved in multiple biological pathways such as regulation of IL-17, JAK-STAT and p53 signaling pathways, etc (**Figures 1C, D**). Additionally, we assessed the mutations for PCD-related genes, revealing that approximately 93.17% (409/439) of GC patients exhibited mutations, predominantly missense mutations (**Figure 1E**). Notably, among the top 20 mutated PCD-related genes, TNN and TP53 exhibited mutation frequencies exceeding 30%. Analysis of CNV status indicated that PCD-related genes frequently underwent alterations. It was noted that the CNV deletion of SLC25A4 was the most extensive, while the copy number amplification of GSDMC was the most significant (**Figure 1F**).

### Prognostic gene signature construction with PCD‐related genes

**Figure 2:**
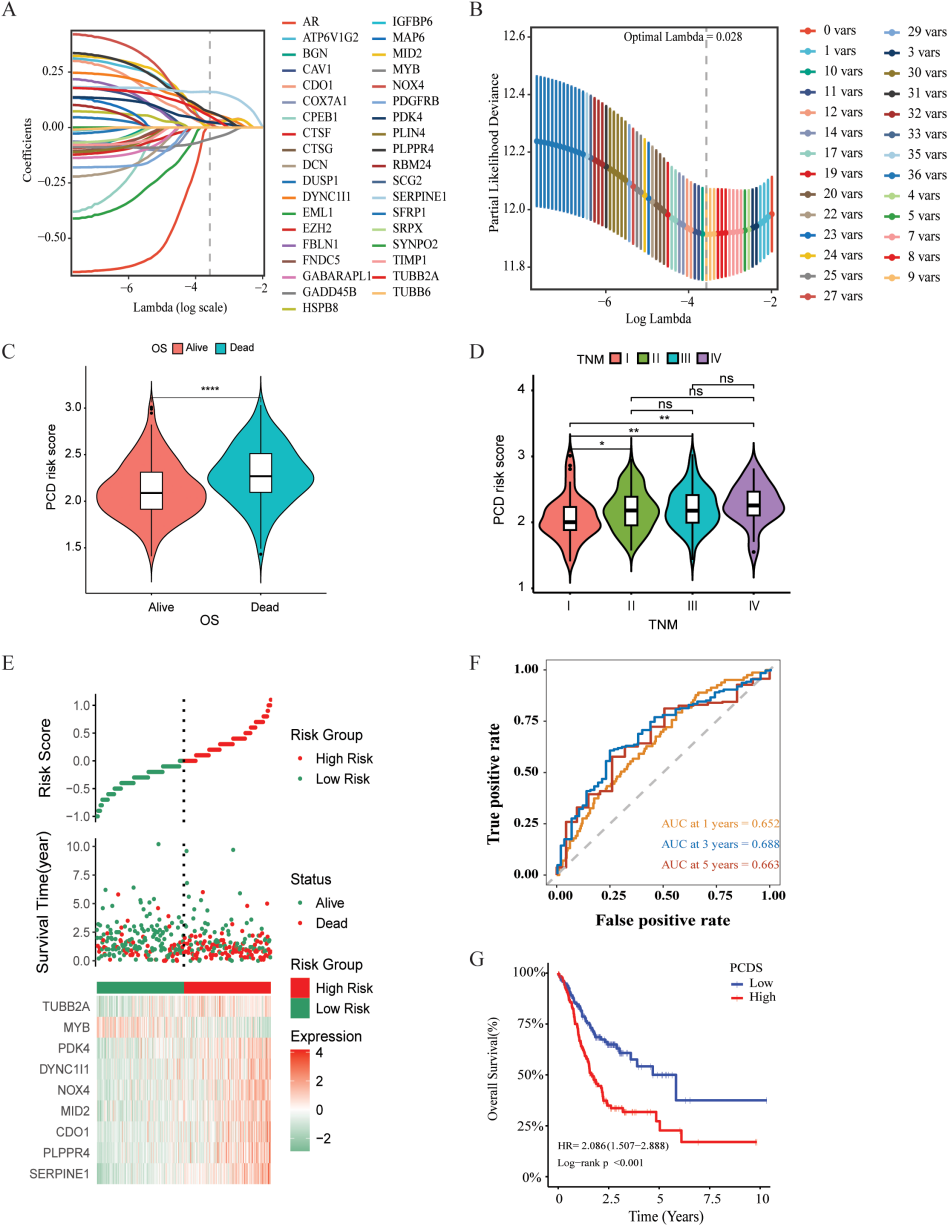
Construction and validation of PCDS signature for GC patients. **(A)** Selection of the model genes by LASSO. **(B)** Cross-validation of the constructed signature. **(C)** Violin plots of the relationship between PCDS and survival status. **(D)**Violin plots of the relationship between PCDS and clinical stage. **(E)** Distribution of PCDS, survival status and time, heatmap of PCDS including 9 genes in TCGA cohorts. **(F)** Kaplan–Meier curves of PCDS predicting the OS of patients in TCGA cohorts. **(G)** Time dependent ROC analysis of the PCDS predicting the OS of patients in TCGA cohorts. **** means P < 0.0001; ** means P < 0.01; * means P < 0.05; ns means not significant.

Survival information of GC patients was collected and subjected to further analysis. Univariate Cox regression analysis was used to screen prognostic-related genes. A total of 80 genes in the TCGA cohort, 74 genes in the GSE84437 cohort, and 59 genes in the GSE84433 reached the cutoff value of P < 0.05 (**Supplementary Tables 4–6**). The intersection of the TCGA and GSE84437 contained 37 genes (See **Supplementary Figure 1**). Then LASSO-Cox regression analysis was applied to further screen the above 37 prognosis-related genes, and the optimal penalty parameter (lambda value λ = 0.028) was selected. Nine genes were screened out, namely: SERPINE1, PLPPR4, CDO1, MID2, NOX4, DYNC1I1, PDK4, MYB, TUBB2A, as shown in **Figures 2A, B**. SERPINE was linked to both apoptosis and cuproptosis. Meanwhile, 4 genes (CDO1, NOX4, PDK4, MYB) were associated with ferroptosis, and 3 genes (MID2, DYNC1I1, TUBB2A) were related to autophagy. Kaplan-Meier analysis revealed that each model gene significantly impacts OS for GC patients (P < 0.05, **Supplementary Figure 2**). PCDS were calculated based on the expression level of each gene and the corresponding correlation Coefficient. The PCDS = 0.1606 *SERPINE1 + 0.0708 *PLPPR4* + 0.0271 * CDO1 + 0.0165* NOX4 + 0.0129 * MID2 + 0.0643 *DYNC1I1 + 0.0242 *PDK4 -0.0522 *MYB + 0.0513 *TUBB2A). The PCDS were significantly associated with survival status (alive or dead) and clinical stage (I-IV) (**Figures 2C, D**). According to the median PCDS value of 2.174, we divided the GC patients in the TCGA cohort into high and low PCDS groups. Prognostic comparison between the groups revealed that individuals with high PCDS exhibited poorer outcomes than those with low PCDS, and the PCA heatmap demonstrated satisfactory classification based on PCDS **Figure 2E**). There was a significant difference in OS between the two groups, the high PCDS group exhibited a higher mortality rate (P < 0.05, **Figure 2G**). The area under the ROC (AUC) values of the PCDS predicting the OS of GC patients at 1 year, 3 years and 5 years were 0.652, 0.688, and 0.663, respectively (**Figure 2F**). Similarly, for the validation cohorts, patients were stratified into high and low PCDS groups based on the median PCDS values of 2.153 for GSE84437 and 2.167 for GSE84433, respectively. Furthermore, comparable robust prognostic performance was observed in the independent cohorts GSE84437 and GSE84433. (**Supplementary Figure 3**).

### Landscape of the tumor immune microenvironment

**Figure 3:**
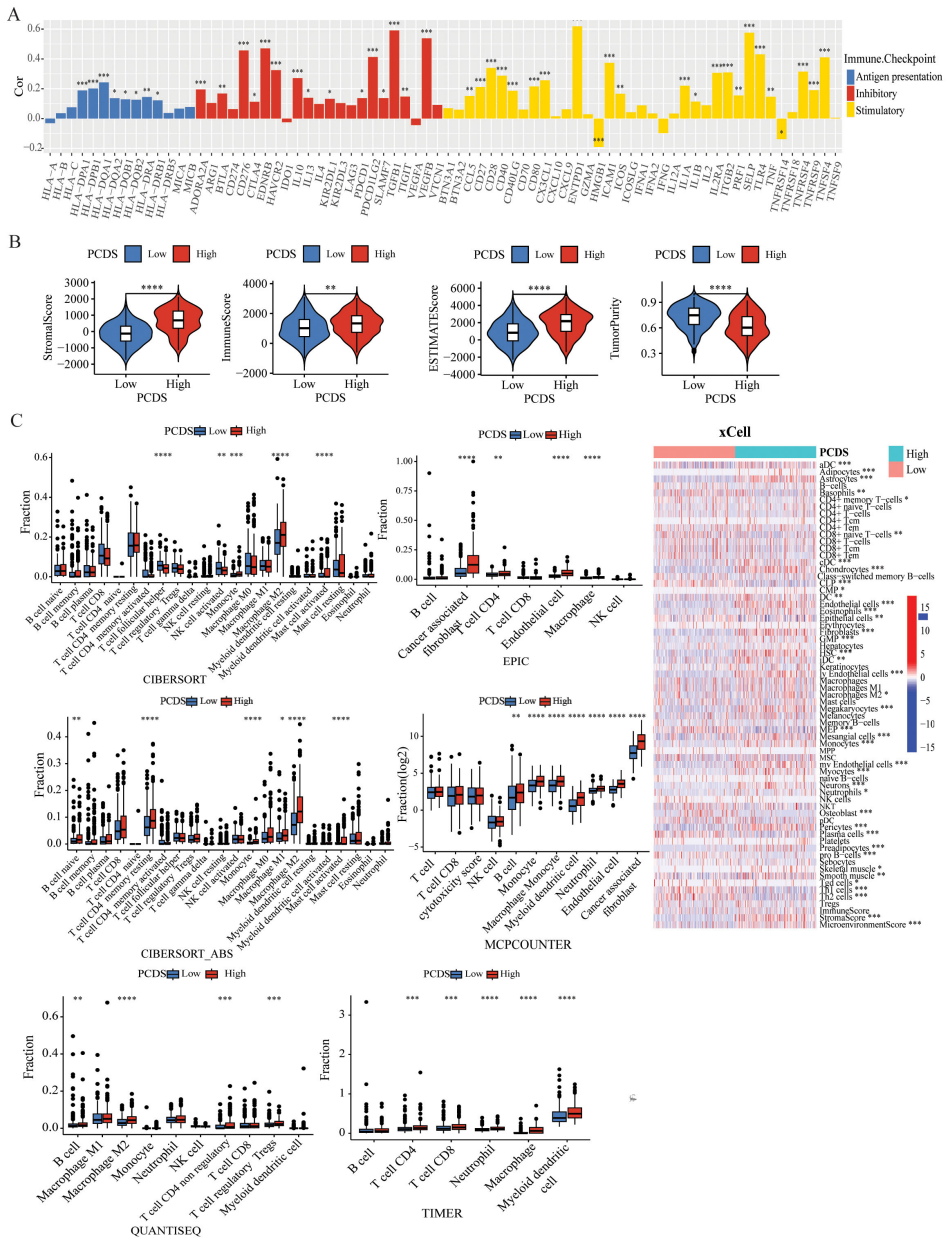
Dissection of TIME based on PCDS signature. **(A)** Bar plot of the correlation between immunomodulators and the PCDS in GC patients. **(B)** Association between PCDS and tumor microenvironment by Estimate algorithms. **(C)** Association between PCDS and stromal and immune infiltration estimations by TIMER, CIBERSORT, quanTIseq, xCell, MCP-counter and EPIC algorithms. **** means P < 0.0001; *** means P < 0.001; ** means P < 0.01; * means P < 0.05.

Increasing evidence indicates that PCDS has a significant impact on the activation of certain anti-tumor immune responses. In this study, we analyzed the composition of the TIME between the PCDS groups. The correlation between PCDS values and immunomodulators in GC patients was analyzed, revealing that higher PCDS values was associated with a higher expression of immune checkpoint-related molecules, indicating an immune regulatory imbalance with high PCDS values (**Figure 3A**). In the ESTIMATE algorithm, we observed a positive correlation between PCDS and the stromal score, immune score, and ESTIMATE score, and a statistically significant negative correlation between PCDS and tumor purity, indicating that PCDS can effectively predict the infiltration levels of stromal cells and immune cells in GC tissues (**Figure 3B**). To confirm this hypothesis, we obtained the abundance of stromal and immune infiltration estimated by algorithms such as CIBERSORT, CIBERSORT-ABS, EPIC, ESTIMATE, MCPCOUNTER, QUANTISEQ, TIMER, and XCELL from the TIMER (http://timer.cistrome.org/) for verification. The results showed that PCDS was positively proportional to the infiltration abundance of stromal cells and immune cells, especially macrophages, Tregs, cancer-associated fibroblasts (CAFs), endothelial cells, etc. (**Figure 3C**). Similar results were also observed in validation cohorts (GSE84437 and GSE84433) (**Supplementary Figure 4**). For further evaluating the effect of the signature on TME, we collected scRNA-seq data from GSE183904 datasets, and clustered subpopulations, and identified marker genes in each cell subtype (**Supplementary Figures 5A, D**). We calculated the PCDS for each sample based on scRNA-seq data (**Supplementary Figures 5B, C**). Samples were divided into two groups (high and low) according to median PCDS. And the proportion of each cell subtype in high and low PCDS groups revealed an increase in mononuclear phagocytes and plasma cells infiltration in the high PCDS group (**Supplementary Figure 5E**). We further sub-grouped the mononuclear phagocytes subtypes and found high proportion of M2 macrophages cells in the high PCDS group, which may suggest that the high PCDS group is correlated with a higher degree of malignancy (**Supplementary Figures 5F–H**).

### Genetic characteristics of different PCDS groups

**Figure 4:**
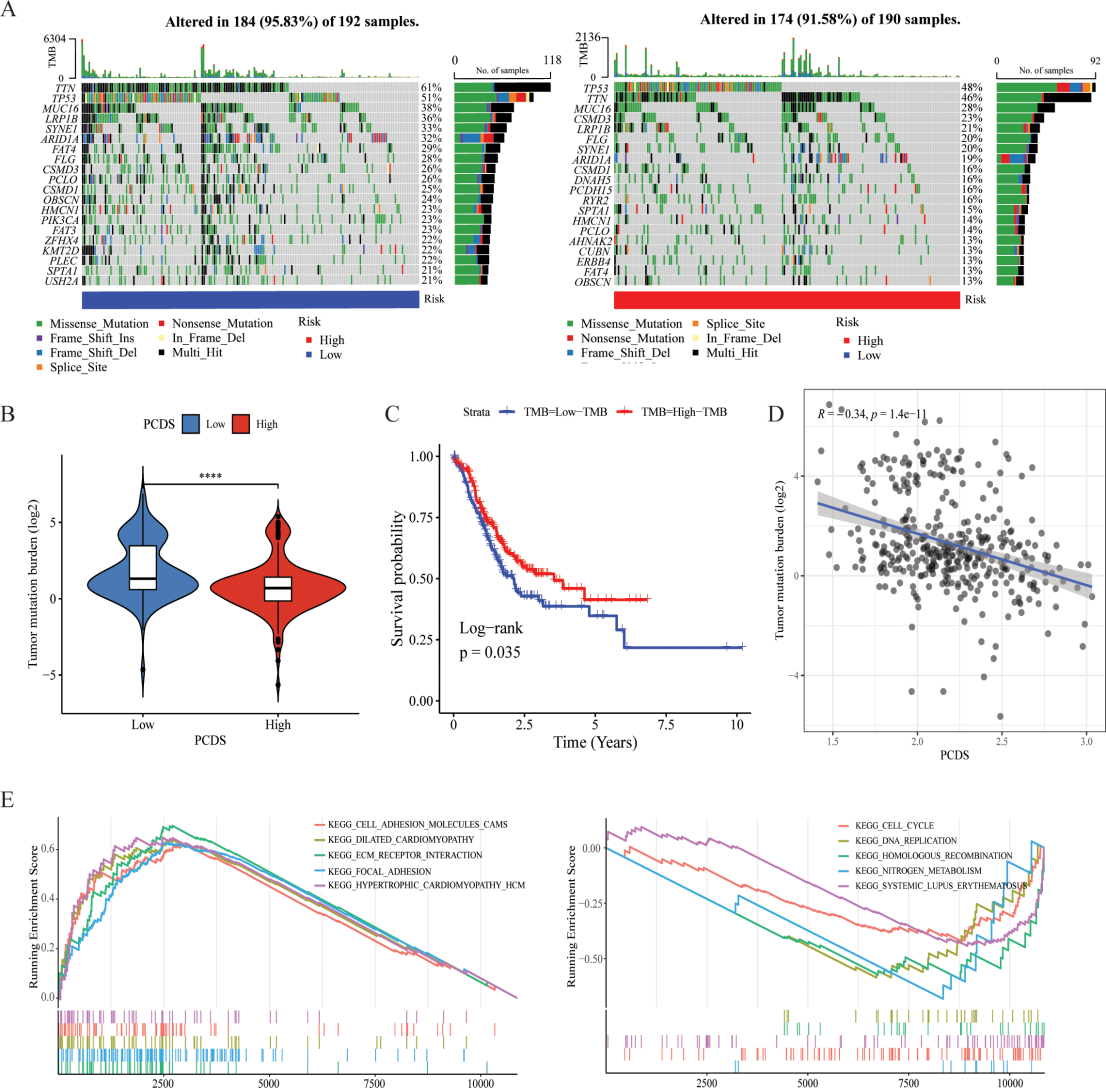
Characteristics of different PCDS groups. **(A)** Genetic mutation landscape in the high- and low PCDS groups. **(B)** Association between TMB and PCDS. **(C)** Kaplan–Meier survival analysis between TMB groups. **(D)** The correlation between the TMB and PCDS in GC patients. **(E)** Representative KEGG pathways upregulated in the high and low PCDS groups. **** means P <0.0001.

Utilizing single nucleotide variant (SNV) data sourced from TCGA-STAD, we compared the variations in TMB between PCDS cohorts. According to the results, we can conclude that the mutation frequency of various genes was higher in the low PCDS group compared to the high PCDS group, such as TTN, LRP1B, CSMD3 and SYNE1 (**Figure 4A**, **Supplementary Table 7**). The results indicated that the TMB in the low PCDS group was substantially higher than in the high PCDS group (**Figure 4B**). TMB exhibited a negative correlation with PCDS, with a correlation coefficient of -0.34 (**Figure 4D**). Additionally, survival analysis revealed that the prognosis for the high TMB group was significantly superior to that of the low TMB group (**Figure 4C**). In the high PCDS group, pathways associated with cancer invasion and metastasis, such as wnt/beta-catenin and cell adhesion molecules, were markedly activated, while pathways involved in DNA damage repair were significantly down-regulated (**Figure 4E**).

### Drug sensitivity analysis between PCDS groups

**Figure 5:**
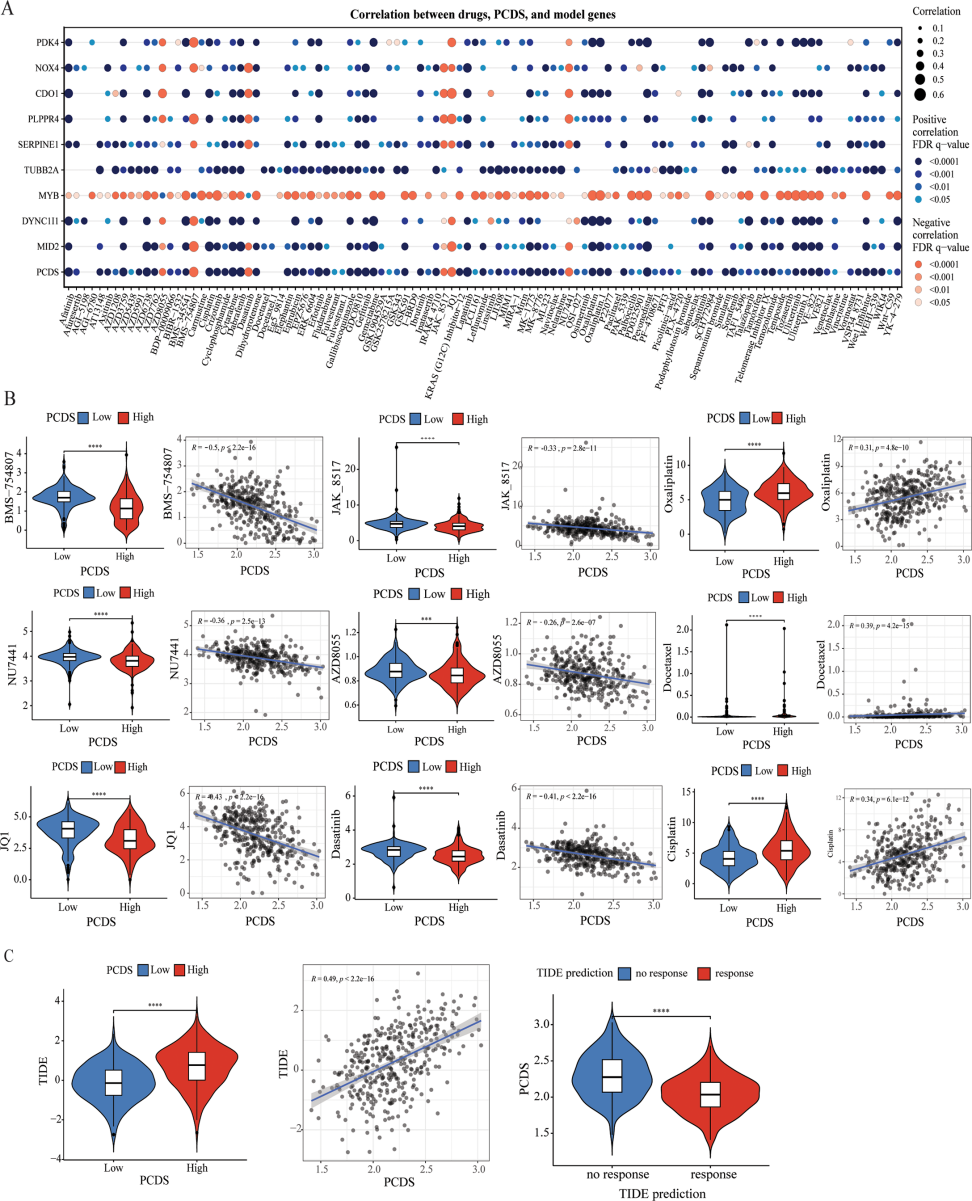
Efficacy of PCDS signature in predicting drug sensitivity. **(A)** Bubble plot of the relationship between drugs, PCDS, and model genes. **(B)** The comparison of IC50 of drugs between high and low PCDS groups, and correlation between the IC50 and PCDS in GC patients. **(C)** The comparison of TIDE score between high and low PCDS groups, and correlation between the TIDE score and PCDS values in GC patients. *** means P < 0.0001; **** means P < 0.0001.

We further investigated the relationship between PCDS and drug sensitivity by comparing the IC50 values of various drugs across PCDS groups. As illustrated in **Figures 5A, B**, IC50 values of most drugs, including traditional chemotherapy regiments such as Cisplatin, Oxaliplatin, and Docetaxel, showed a positive correlation with PCDS, indicating that patients with high PCDS were generally insensitive to them. Conversely, six drugs—NU7441, AZD8055, Dasatinib, JAK_8517, BMS-754807, and JQ1—exhibited a significantly negative correlation with PCDS, suggesting potential efficacy in patients with high PCDS. Furthermore, an evaluation of the TIDE scores for each GC patient revealed a marked increase in scores within the high PCDS group, implying poor efficacy of ICIs therapy (**Figure 5C**). Additionally, regarding immunotherapy response, PCDS values were lower in the response group, indicating that patients with low PCDS might derive greater benefit from immunotherapy, whereas those with high PCDS may not (**Figure 5C**).

### Establishment and assessment of the nomogram survival model

**Figure 6:**
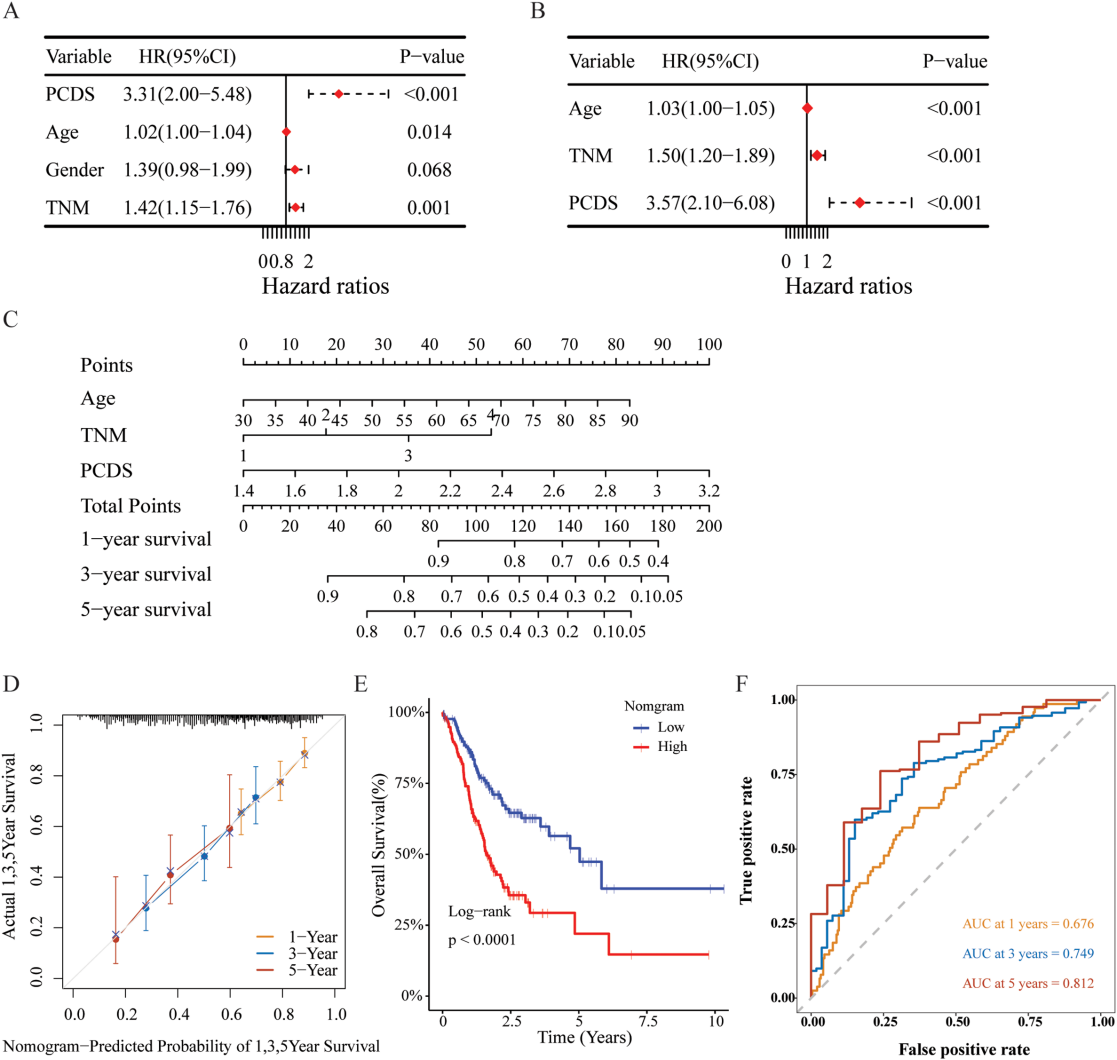
Construction and assessment of the nomogram survival model. **(A, B)** Univariate and multivariate analysis of PCDS and the clinicopathologic characteristics. **(C)** A nomogram was established to predict the prognostic of GBM patients. **(D)** The calibration curve of the nomogram in TCGA cohort. **(E)** Kaplan-Meier analyses for the two groups based on the nomogram score in in TCGA cohort. **(F)** Receiver operator characteristic (ROC) analysis of nomogram in TCGA cohorts.

Univariate and multivariate Cox regression analyses were conducted to assess whether PCDS serves as an independent prognostic indicator. The univariate Cox regression analysis revealed that, compared with other characteristics, PCDS was a significant risk factor (HR=3.31, 95% CI 2.00-5.48, P < 0.001, **Figure 6A**). After adjusting for confounding factors, the multivariate analysis confirmed that PCDS remained an independent prognostic factor for GC patients (HR =3.57, 95% CI 2.10-6.08, P < 0.001, **Figure 6B**). Then, a nomogram model was established in the TCGA cohort using multivariate Cox and stepwise regression analyses to estimate 1-year, 3-year, and 5-year OS. Age, TNM stage, and PCDS were incorporated into the nomogram model (**Figure 6C**). Delong’s test demonstrated that the C-index value of the nomogram (0.653, 95% CI: 0.608-0.698) was significantly higher than that of the TNM stage (0.574, 95% CI:0.525–0.623) (**Supplementary Table 8**, P < 0.05). The calibration curve showed the nomogram’s satisfactory accuracy in predicting 1-year, 3-year, and 5-year OS (**Figure 6D**). According to the nomogram score, there was a significant difference in OS between the high and low nomogram score groups (**Figure 6E**). Furthermore, ROC curve analysis revealed that the AUC values of the nomogram for prognostic performance of GC patients at 1 year, 3 years, and 5 years were 0.676, 0.749, and 0.812, respectively (**Figure 6F**). And the findings also demonstrated that the nomogram exhibited higher prognostic accuracy than traditional TNM stage, PCDS, and age alone (**Supplementary Figure 7**). Additionally, external validation in the GSE844437 and GSE84433 cohorts further confirmed its satisfactory performance (**Supplementary Figures 6, 7**).

### Immunohistochemical analysis

We further validated the expression of PCDS-related genes in gastric cancer and normal tissues using IHC. SERPINE1 and MYB expressions were lower in normal gastric tissues than in gastric cancers, while PDK4 and TUBB2A were higher in normal tissues compared to GC patients (**Supplementary Figure 8**). CDO1 protein expression was not detected in gastric cancers. Information on PLPPR4, NOX4, MID2, and DYNC1I1 expression in GC was unavailable in the HPA database. However, studies indicate that NOX4, MID2, and DYNC1I1 are significantly elevated in GC tissues or other cancers compared to normal tissues, as determined by immunohistochemistry (45–47).

## Discussion

This investigation presents an extensive preliminary analysis of 14 different PCD patterns in GC. The study involved the construction of a PCDS signature within the TCGA-STAD cohort, subsequently validated by the GSE84437 and GSE84433 cohorts, affirming its robust efficacy. A nomogram model incorporating clinical characteristics and PCDS was developed, yielding promising outcomes for predicting OS for GC patients. Furthermore, the study examined potential associations between PCDS and TIME via various methodologies, suggesting implications for immunotherapy strategies in GC. Additionally, the correlation between PCDS and drug responsiveness was assessed, revealing that patients with a high PCDS may exhibit resistance to immunotherapy and standard adjuvant chemotherapy regimens; however, they may benefit from drugs such as NU7441, AZD8055, Dasatinib, JAK-, BMS-754807, and JQ1, with “Dasatinib” being an FDA-approved medication. The development of this comprehensive PCDS signature enhances the understanding of the intricate biological processes underlying GC. It provides a valuable tool for evaluating the prognosis of GC patients and guiding treatment decisions. Integrating PCDS into prognostic models offers promising prospects for personalized medicine, enabling the development of tailored treatment strategies for individual patients.

According to reports, SERPINE promotes malignant progression and correlates with poor prognosis in GC (48). Teng et al. found that the NKX2-1-AS1/miR-145-5p axis induces SERPINE1 translation, thus activating the VEGFR-2 signaling pathway to promote tumor progression and angiogenesis in GC (49). PLPPR4, also named LPPR4, Zhang et al. found that LPPR4 could promote the migration, invasion and adhesion of GC cells to facilitate peritoneal metastasis through the Sp1/integrin α/FAK pathway (50). CDO1 possesses functionally oncogenic aspects through modification of mitochondrial membrane potential (51). Mouse experiments have revealed that inhibiting CDO1 production facilitates ferroptosis by increasing oxidative stress and inhibiting GPX4 production (52). Harada et al.’s study suggested that abnormal CDO1 expression in GC may indicate distant metastatic ability (53). Current investigations into the mechanistic role of MID2 in GC remain limited. Nonetheless, MID2 inhibition could largely abrogate MORC4-induced drug resistance to adriamycin, 5-fluorouracil, and cisplatin in breast cancer (54). Abnormal NOX4 expression results in the production of ROS, contributing to various oncogenic processes (55). Tang et al. found that NOX4 promotes GC cell growth and apoptosis through the generation of ROS and subsequent activation of GLI1 signaling (56). DYNC1I1, as an important binding subunit of cytoplasmic dynein, primarily participates in cell cycle regulation (57). Gong et al. revealed that DYNC1I1 could upregulate IL-6 expression by increasing NF-κB nuclear translocation, and then trigger the DYNC1I1-driven IL-6/STAT pathway to promote GC proliferation and migration (47). High PDK4 expression is closely related to poor prognosis, and might participate in the proliferation, migration and invasion of GC cells by modulating the glycolysis level in GC cells (58). Miao et al. discovered that miR-5683 represses GC glycolysis and progression through targeting PDK4 (59). Furthermore, high MYB expression is positively associated with activated CD4+ T cell infiltration and poor prognosis in GC (60). Yan et al. demonstrated that SNHG3 binds and sequesters miR-139-5p, which can indirectly promote the upregulation of the miR-139-5p target gene MYB and drive the proliferation, migration, and invasion in GC (61). Additionally, high TUBB2A expression is linked to reduced immune cell infiltration and poor prognosis in triple-negative breast cancer (62).

According to prior research, the tumor microenvironment plays crucial roles in tumor initiation, progression, metastasis, and response to therapies. Moreover, tumor cells can survive because the tumor microenvironment allows them to evade immune surveillance and drug interference (63, 64). In this study, PCDS was correlated with the infiltration abundance of stromal and immune cells, notably M2 macrophages, Tregs, CAFs, and endothelial cells. Among these, M2 macrophages, also referred to as tumor-associated macrophages, facilitate tumor progression by fostering cancer invasion and metastasis, promoting neovascularization, and contributing to the development of an immunosuppressive TME (65). Endothelial cells mainly provide nutrition for tumor development. By responding to angiogenic factors such as VEGF, they promote new blood vessel formation and provide oxygen and nutrition for tumors, and playing a key role in the angiogenesis of gastric cancer (66). CAFs induce hypoxia in the tumor microenvironment, leading to ECM hardening and degradation, which in turn affects tumor cell proliferation, migration and invasion as well as angiogenesis (67). Tregs can be divided into two types: natural regulatory T cells (nTregs) and induced regulatory T cells (iTregs) (68). nTregs originate from the thymus and play a role in mediating immune tolerance through transcription factors such as nuclear factor κB (NF-κB), while iTregs develop in the peripheral environment and are stimulated by inhibitory cytokines IL-2 and TGF-β in the tumor microenvironment, which in turn helps GC cells evade immune surveillance (69). Tregs regulate immune cell activity in the tumor microenvironment, suppressing cytotoxic T and natural killer cells, reducing immune responses, enabling tumor cells to evade immune surveillance, and fostering a tumor growth-permissive environment (70). Increasing evidence indicates that high TMB can send signals to activate immune responses, thereby making tumors more sensitive to immunotherapy (71). GC patients with high TMB exhibit superior OS compared to those with low TMB (72). These findings align with our results. Our results showed that TMB was significantly negatively correlated with PCDS. In terms of molecular pathways, compared with the low PCDS group, cancer-related pathways such as Wnt/beta-catenin and cell adhesion molecules were overactivated in the high PCDS group. Therefore, immunotherapy may be an effective treatment approach for patients with low PCDS, while those with high PCDS may not benefit as much.

Drug sensitivity analysis indicates that GC patients with high PCDS may exhibit resistance to immunotherapy and standard adjuvant chemotherapy regimen. Notably, PCDS showed a significant negative correlation with the IC50 values of NU7441, AZD8055, dasatinib, JAK-8517, BMS-754807, and JQ1, implying these drugs may have potential benefits for GC patients with high PCDS. BMS-754807, a selective IGF-1R inhibitor, exhibits potent inhibitory effects on GC cells (73). Another study also demonstrated the activation of the IGF1/IGF1R pathway in mesenchymal gastric tumors, which showed sensitivity to another selective IGF-1R inhibitor, Linsitinib (OSI-906) (74). Likewise, mTOR inhibitors such as 2,6-DMBQ (AZD8055) have also been previously reported for their inhibitory efficacy in GC (75). AZD8055 inhibits the phosphorylation of mTORC1 substrates p70S6K and 4E-BP1 as well as phosphorylation of the mTORC2 substrate AKT and downstream proteins, thereby leading to tumor growth inhibition (76). Dasatinib plays a synergistic role with oxaliplatin in inhibiting gastric cancer cell growth both *in vitro* and *in vivo*, via inhibiting Src activity stimulated by oxaliplatin (77). Wang Shi et al. found dasatinib also showed potential in sensitizing cancer cells to cisplatin, and the PI3K/AKT pathway was involved in the anti-cancer effect of dasatinib or combined with cisplatin (78). In addition, dasatinib is FDA-approved drug. BET protein inhibitor JQ1 downregulates chromatin accessibility and suppresses metastasis of gastric cancer via inactivating RUNX2/NID1 signaling (79). Furthermore, JQ1 augments the antitumor efficacy of abemaciclib (ABE) in preclinical models of gastric carcinoma. Mechanistically, the combination of ABE and JQ1 enhances the cell cycle arrest of GC cells and induces unique characteristics of cellular senescence through the induction of DNA damage (80). NU7441, a DNA−PKcs inhibitor, increases the sensitivity of GC cells to radiotherapy (81). This inhibitor increases the sensitivity of radioresistant BGC823 and MGC803 cells to radiotherapy through the cleaved−caspase3/γH2AX signaling pathway, thus presenting a potential treatment method for GC (81). JAK_8517 is a small molecule inhibitor that targets Janus kinase (JAK), which is involved in cell signaling. Thus, GC patients with high PCDS patients may benefit from the above six candidate drugs, especially the FDA-approved drug dasatinib.

Although our model has demonstrated excellent performance in both the training and validation cohorts, it is important to acknowledge certain limitations. First, retrospective recruitment of patients may introduce some inherent biases to a certain extent. Second, more experiments are needed. Therefore, additional validation through high-quality, multicenter randomized controlled trials with sufficient sample sizes and adequate follow-up is necessary.

## Conclusion

In conclusion, through a comprehensive analysis of 14 PCD pattern-related genes, a new PCDS signature has been established. This innovative signature accurately predicts the prognosis and drug sensitivity of GC. The findings indicated that PCDS can serve as a valuable tool for evaluating the prognosis and guiding immunotherapy treatment decisions for GC patients.

## Data Availability

The original contributions presented in the study are included in the article/**Supplementary Material**. Further inquiries can be directed to the corresponding author.
