# Supplementary material for: Multiple programmed cell death patterns predict the prognosis and drug sensitivity in gastric cancer
Source: Front Immunol. 2025 Feb 4;16:1511453. doi: 10.3389/fimmu.2025.1511453 (PMC11832517; doi:10.3389/fimmu.2025.1511453)
Supplement: Supplementary file 2 [file Table2.docx]

Supplementary Table 2. The clinical information of three independent cohorts.

|  | TCGA | GSE84437 | GSE84433 |
| --- | --- | --- | --- |
| Survival status |  |  |  |
| alive | 226 | 224 | 183 |
| dead | 159 | 209 | 174 |
| Age (mean (SD)) | 64.77 (10.52) | 60.06 (11.58) | 59.52 (11.31) |
| Gender |  |  |  |
| female | 127 | 137 | 115 |
| male | 228 | 296 | 242 |
| T stage |  |  |  |
| T1 | 17 | 11 | 11 |
| T2 | 74 | 38 | 35 |
| T3 | 166 | 92 | 67 |
| T4 | 9 | 292 | 244 |
| N stage |  |  |  |
| N0 | 110 | 80 | 71 |
| N1 | 98 | 188 | 155 |
| N2 | 73 | 132 | 99 |
| N3 | 74 | 33 | 32 |
| M stage |  |  |  |
| M0 | 331 |  |  |
| M1 | 24 |  |  |
| TNM stage |  |  |  |
| I | 47 | 21 | 21 |
| II | 129 | 91 | 71 |
| III | 155 | 321 | 265 |
| IV | 24 |  |  |

Supplementary Table 4. Univariate Cox regression analysis of PCD-related gene in TCGA cohort.

|  | gene | HR | z | pvalue | lower | upper |
| --- | --- | --- | --- | --- | --- | --- |
| 1 | SERPINE1 | 1.318256 | 4.196021 | 2.72E-05 | 1.158638 | 1.499864 |
| 2 | GPX3 | 1.350021 | 4.042665 | 5.28E-05 | 1.16721 | 1.561465 |
| 3 | PLPPR4 | 1.379874 | 3.495245 | 0.000474 | 1.151925 | 1.652931 |
| 4 | CDO1 | 1.353226 | 3.407427 | 0.000656 | 1.137118 | 1.610404 |
| 5 | FABP4 | 1.206106 | 3.395047 | 0.000686 | 1.082434 | 1.343908 |
| 6 | MID2 | 1.410562 | 3.355391 | 0.000793 | 1.153795 | 1.72447 |
| 7 | GRIA3 | 2.198204 | 3.34914 | 0.000811 | 1.386389 | 3.485387 |
| 8 | NOX4 | 1.380442 | 3.248948 | 0.001158 | 1.136451 | 1.676817 |
| 9 | ELANE | 1.321106 | 3.226793 | 0.001252 | 1.115526 | 1.564571 |
| 10 | MRGPRX2 | 2.563591 | 3.206568 | 0.001343 | 1.441938 | 4.557756 |
| 11 | HSPB8 | 1.180968 | 3.205178 | 0.00135 | 1.066755 | 1.30741 |
| 12 | CAV1 | 1.218365 | 3.179307 | 0.001476 | 1.078693 | 1.376123 |
| 13 | DUSP1 | 1.286506 | 3.166002 | 0.001545 | 1.100725 | 1.503643 |
| 14 | DYNC1I1 | 1.283932 | 3.110494 | 0.001868 | 1.096853 | 1.50292 |
| 15 | RNASE1 | 1.246709 | 3.09109 | 0.001994 | 1.084035 | 1.433794 |
| 16 | MAPK10 | 1.822968 | 2.99241 | 0.002768 | 1.230196 | 2.701368 |
| 17 | GADD45B | 1.389393 | 2.991032 | 0.00278 | 1.120043 | 1.723516 |
| 18 | MAP6 | 1.399965 | 2.968564 | 0.002992 | 1.121101 | 1.748193 |
| 19 | PDK4 | 1.169355 | 2.949558 | 0.003182 | 1.053893 | 1.297467 |
| 20 | TUBB6 | 1.304178 | 2.934406 | 0.003342 | 1.092196 | 1.557303 |
| 21 | PER1 | 1.345139 | 2.919512 | 0.003506 | 1.102355 | 1.641394 |
| 22 | SRPX | 1.179381 | 2.913429 | 0.003575 | 1.05548 | 1.317827 |
| 23 | FBLN1 | 1.163049 | 2.888387 | 0.003872 | 1.049749 | 1.288577 |
| 24 | SFRP1 | 1.139265 | 2.868406 | 0.004125 | 1.042158 | 1.245421 |
| 25 | TUBB4A | 1.38443 | 2.856343 | 0.004286 | 1.107474 | 1.730646 |
| 26 | F2R | 1.421887 | 2.828283 | 0.00468 | 1.114119 | 1.814675 |
| 27 | PIM1 | 1.369102 | 2.816191 | 0.00486 | 1.100225 | 1.703688 |
| 28 | MYB | 0.827411 | -2.75309 | 0.005904 | 0.723012 | 0.946883 |
| 29 | PTGIS | 1.159135 | 2.733586 | 0.006265 | 1.042678 | 1.288598 |
| 30 | BGN | 1.246574 | 2.706901 | 0.006791 | 1.062704 | 1.462257 |
| 31 | AADAC | 1.140614 | 2.700831 | 0.006917 | 1.036749 | 1.254885 |
| 32 | DCN | 1.1916 | 2.690201 | 0.007141 | 1.048733 | 1.353929 |
| 33 | GABARAPL1 | 1.331043 | 2.674916 | 0.007475 | 1.079429 | 1.641308 |
| 34 | SNCA | 1.272444 | 2.671706 | 0.007547 | 1.066291 | 1.518453 |
| 35 | IGFBP6 | 1.225746 | 2.64248 | 0.00823 | 1.053981 | 1.425503 |
| 36 | FGF10 | 1.289451 | 2.594755 | 0.009466 | 1.064167 | 1.562427 |
| 37 | MAP1LC3C | 1.56806 | 2.557103 | 0.010555 | 1.110763 | 2.213625 |
| 38 | ATP6V1G2 | 1.646784 | 2.553383 | 0.010668 | 1.122916 | 2.415049 |
| 39 | PDGFRB | 1.244795 | 2.548264 | 0.010826 | 1.051852 | 1.473129 |
| 40 | MTUS2 | 2.028829 | 2.543223 | 0.010984 | 1.176151 | 3.499674 |
| 41 | KIT | 1.207631 | 2.528408 | 0.011458 | 1.043328 | 1.397809 |
| 42 | ZFP36 | 1.29999 | 2.524706 | 0.01158 | 1.060442 | 1.59365 |
| 43 | SCG2 | 1.16703 | 2.508498 | 0.012125 | 1.034353 | 1.316726 |
| 44 | PYGM | 1.278186 | 2.486281 | 0.012909 | 1.053331 | 1.551042 |
| 45 | ANGPTL7 | 1.338094 | 2.472706 | 0.013409 | 1.062254 | 1.685563 |
| 46 | CTSF | 1.217917 | 2.465414 | 0.013686 | 1.041245 | 1.424565 |
| 47 | TSC22D3 | 1.240928 | 2.459326 | 0.01392 | 1.044805 | 1.473867 |
| 48 | PLIN4 | 1.121844 | 2.44237 | 0.014591 | 1.022969 | 1.230276 |
| 49 | PRKAA2 | 1.252373 | 2.429076 | 0.015137 | 1.044419 | 1.501734 |
| 50 | MTTP | 1.138701 | 2.401858 | 0.016312 | 1.024185 | 1.266022 |
| 51 | NRG2 | 1.583534 | 2.39395 | 0.016668 | 1.086899 | 2.307096 |
| 52 | CCNA1 | 1.626431 | 2.392254 | 0.016745 | 1.091871 | 2.422702 |
| 53 | CYSLTR1 | 1.32019 | 2.383279 | 0.017159 | 1.050576 | 1.658998 |
| 54 | NR4A3 | 1.284048 | 2.382738 | 0.017184 | 1.04536 | 1.577236 |
| 55 | SOCS2 | 1.270958 | 2.379505 | 0.017336 | 1.043181 | 1.54847 |
| 56 | SYNPO2 | 1.113776 | 2.375525 | 0.017524 | 1.019029 | 1.217332 |
| 57 | MAGEA3 | 1.109381 | 2.367786 | 0.017895 | 1.018039 | 1.208918 |
| 58 | INHBA | 1.272683 | 2.345056 | 0.019024 | 1.040391 | 1.556839 |
| 59 | TIMP1 | 1.283242 | 2.34314 | 0.019122 | 1.041626 | 1.580903 |
| 60 | GRID2 | 2.590495 | 2.328407 | 0.01989 | 1.162554 | 5.772349 |
| 61 | AGT | 1.14415 | 2.328019 | 0.019911 | 1.021518 | 1.281503 |
| 62 | NTN1 | 1.192982 | 2.316982 | 0.020505 | 1.027563 | 1.385031 |
| 63 | FNDC5 | 1.228358 | 2.309272 | 0.020928 | 1.031601 | 1.462644 |
| 64 | CTSG | 1.142043 | 2.293265 | 0.021833 | 1.019491 | 1.279327 |
| 65 | CPEB1 | 1.716147 | 2.247725 | 0.024594 | 1.071589 | 2.748404 |
| 66 | EML1 | 1.18988 | 2.2354 | 0.025391 | 1.021652 | 1.385808 |
| 67 | COX7A1 | 1.187441 | 2.22062 | 0.026377 | 1.020371 | 1.381867 |
| 68 | CNR1 | 1.214388 | 2.210641 | 0.027061 | 1.02227 | 1.442611 |
| 69 | EPM2A | 1.495931 | 2.201243 | 0.027719 | 1.045134 | 2.141168 |
| 70 | EZH2 | 0.766581 | -2.17066 | 0.029957 | 0.603006 | 0.974529 |
| 71 | RBM24 | 1.263346 | 2.167586 | 0.03019 | 1.022644 | 1.560703 |
| 72 | BBC3 | 0.756462 | -2.14123 | 0.032256 | 0.585916 | 0.97665 |
| 73 | AR | 1.349022 | 2.128564 | 0.03329 | 1.023997 | 1.777212 |
| 74 | HBB | 1.123468 | 2.065978 | 0.038831 | 1.005992 | 1.254663 |
| 75 | CXCL12 | 1.158286 | 2.04493 | 0.040862 | 1.006124 | 1.333461 |
| 76 | NRG3 | 1.466341 | 2.014618 | 0.043945 | 1.010438 | 2.127945 |
| 77 | ADRA1A | 1.777956 | 2.013468 | 0.044065 | 1.015409 | 3.113157 |
| 78 | ANGPTL4 | 1.174165 | 1.987633 | 0.046852 | 1.002238 | 1.375586 |
| 79 | KCNN3 | 1.290395 | 1.979978 | 0.047706 | 1.00258 | 1.660833 |
| 80 | TUBB2A | 1.20788 | 1.963644 | 0.049571 | 1.000354 | 1.458458 |

Supplementary Table 5. Univariate Cox regression analysis of PCD-related gene in GSE84437 cohort.

|  | gene | HR | z | pvalue | lower | upper |
| --- | --- | --- | --- | --- | --- | --- |
| 1 | TUBB6 | 1.371493 | 4.352847 | 1.34E-05 | 1.18965 | 1.581132 |
| 2 | IFNG | 0.742407 | -3.90285 | 9.51E-05 | 0.639264 | 0.862192 |
| 3 | SYNPO2 | 1.144159 | 3.800953 | 0.000144 | 1.067401 | 1.226435 |
| 4 | PLIN4 | 1.145532 | 3.70412 | 0.000212 | 1.066068 | 1.23092 |
| 5 | AKR1C2 | 1.152196 | 3.689331 | 0.000225 | 1.068662 | 1.24226 |
| 6 | BRCA1 | 0.688968 | -3.65506 | 0.000257 | 0.564204 | 0.841322 |
| 7 | HSPB8 | 1.159919 | 3.605668 | 0.000311 | 1.070055 | 1.25733 |
| 8 | PDK4 | 1.179565 | 3.549953 | 0.000385 | 1.076772 | 1.292172 |
| 9 | SOX15 | 1.22819 | 3.530538 | 0.000415 | 1.095747 | 1.376642 |
| 10 | FNDC5 | 1.296842 | 3.526498 | 0.000421 | 1.122397 | 1.498399 |
| 11 | CAV1 | 1.218583 | 3.509499 | 0.000449 | 1.091207 | 1.360827 |
| 12 | GSN | 1.292739 | 3.464199 | 0.000532 | 1.117945 | 1.494862 |
| 13 | CDC25A | 0.745152 | -3.40374 | 0.000665 | 0.629042 | 0.882693 |
| 14 | BGN | 1.2542 | 3.380592 | 0.000723 | 1.099858 | 1.4302 |
| 15 | IGFBP6 | 1.223859 | 3.319202 | 0.000903 | 1.086242 | 1.378911 |
| 16 | CTSF | 1.250436 | 3.250216 | 0.001153 | 1.092775 | 1.430843 |
| 17 | NOX4 | 1.3154 | 3.244799 | 0.001175 | 1.114662 | 1.552289 |
| 18 | COX7A1 | 1.220315 | 3.22674 | 0.001252 | 1.081304 | 1.377197 |
| 19 | TUBB3 | 1.274831 | 3.117338 | 0.001825 | 1.094338 | 1.485093 |
| 20 | DYNC1I1 | 1.216825 | 3.113749 | 0.001847 | 1.075427 | 1.376813 |
| 21 | CDO1 | 1.267802 | 3.102944 | 0.001916 | 1.091338 | 1.472799 |
| 22 | BRCA2 | 0.68792 | -3.03999 | 0.002366 | 0.540498 | 0.875551 |
| 23 | STAT1 | 0.789665 | -3.01662 | 0.002556 | 0.677345 | 0.920612 |
| 24 | TPD52L1 | 1.203148 | 3.011329 | 0.002601 | 1.0667 | 1.35705 |
| 25 | HELLS | 0.759997 | -2.97798 | 0.002902 | 0.634407 | 0.910449 |
| 26 | GBP5 | 0.853176 | -2.96981 | 0.00298 | 0.768293 | 0.947438 |
| 27 | RHOB | 1.228024 | 2.921829 | 0.00348 | 1.069958 | 1.409441 |
| 28 | MID2 | 1.286736 | 2.920548 | 0.003494 | 1.086455 | 1.523938 |
| 29 | CHEK2 | 0.724117 | -2.90721 | 0.003647 | 0.5825 | 0.900164 |
| 30 | VLDLR | 1.295135 | 2.873504 | 0.004059 | 1.085693 | 1.54498 |
| 31 | PDGFRB | 1.221221 | 2.85325 | 0.004327 | 1.064568 | 1.400926 |
| 32 | EML1 | 1.194734 | 2.842569 | 0.004475 | 1.056799 | 1.350673 |
| 33 | EMP1 | 1.213359 | 2.826929 | 0.0047 | 1.061104 | 1.38746 |
| 34 | ADGRE2 | 0.768594 | -2.79656 | 0.005165 | 0.639127 | 0.924285 |
| 35 | FANCD2 | 0.752517 | -2.73528 | 0.006233 | 0.613811 | 0.922569 |
| 36 | SRPX | 1.141084 | 2.723442 | 0.006461 | 1.037691 | 1.254778 |
| 37 | MYB | 0.869673 | -2.69568 | 0.007025 | 0.785712 | 0.962607 |
| 38 | EZH2 | 0.780008 | -2.69437 | 0.007052 | 0.651042 | 0.934522 |
| 39 | RBM24 | 1.274982 | 2.57671 | 0.009975 | 1.059871 | 1.533753 |
| 40 | CPEB1 | 1.507796 | 2.560885 | 0.010441 | 1.101156 | 2.064603 |
| 41 | TP53INP2 | 1.271927 | 2.527526 | 0.011487 | 1.055498 | 1.532736 |
| 42 | TMEM74 | 1.690782 | 2.515067 | 0.011901 | 1.122901 | 2.545857 |
| 43 | SFRP1 | 1.096183 | 2.510746 | 0.012048 | 1.02035 | 1.177652 |
| 44 | STMN1 | 0.768746 | -2.47834 | 0.013199 | 0.624389 | 0.946477 |
| 45 | DCN | 1.162717 | 2.470982 | 0.013474 | 1.031669 | 1.310411 |
| 46 | TUBB2A | 1.219852 | 2.47007 | 0.013509 | 1.041894 | 1.428205 |
| 47 | TNFRSF10B | 0.765643 | -2.44679 | 0.014413 | 0.618198 | 0.948255 |
| 48 | SCG2 | 1.145212 | 2.441205 | 0.014638 | 1.02709 | 1.27692 |
| 49 | TMEM238L | 0.852378 | -2.42454 | 0.015328 | 0.749129 | 0.969858 |
| 50 | RRM2 | 0.848191 | -2.41385 | 0.015785 | 0.74205 | 0.969515 |
| 51 | ATP6V1G2 | 1.414919 | 2.360684 | 0.018241 | 1.060685 | 1.887456 |
| 52 | ZNF385B | 1.216231 | 2.358858 | 0.018331 | 1.033657 | 1.431053 |
| 53 | SLC25A4 | 1.18334 | 2.316599 | 0.020526 | 1.026254 | 1.36447 |
| 54 | FIGNL1 | 0.764659 | -2.30813 | 0.020992 | 0.608855 | 0.960333 |
| 55 | MAP6 | 1.225395 | 2.279655 | 0.022628 | 1.028915 | 1.459394 |
| 56 | DUSP1 | 1.167066 | 2.273098 | 0.02302 | 1.02151 | 1.333362 |
| 57 | SERPINE1 | 1.129497 | 2.264102 | 0.023568 | 1.016492 | 1.255065 |
| 58 | FBLN1 | 1.112364 | 2.262564 | 0.023663 | 1.014344 | 1.219857 |
| 59 | GADD45B | 1.2209 | 2.259742 | 0.023837 | 1.026831 | 1.451647 |
| 60 | GABARAPL1 | 1.215608 | 2.238013 | 0.02522 | 1.024554 | 1.442288 |
| 61 | RUFY4 | 0.644618 | -2.17881 | 0.029345 | 0.434269 | 0.956853 |
| 62 | CDC25B | 0.849294 | -2.15602 | 0.031082 | 0.732095 | 0.985255 |
| 63 | PARP14 | 0.821516 | -2.14538 | 0.031923 | 0.686454 | 0.983152 |
| 64 | PLPPR4 | 1.192571 | 2.14297 | 0.032115 | 1.015153 | 1.400996 |
| 65 | LEF1 | 1.170744 | 2.119359 | 0.03406 | 1.011926 | 1.354487 |
| 66 | GPER1 | 1.139569 | 2.083742 | 0.037184 | 1.007791 | 1.288578 |
| 67 | LAMP3 | 0.870236 | -2.0802 | 0.037507 | 0.76342 | 0.991998 |
| 68 | TIMP1 | 1.191666 | 2.076699 | 0.037829 | 1.009906 | 1.40614 |
| 69 | MELK | 0.872558 | -2.0762 | 0.037875 | 0.76719 | 0.992397 |
| 70 | TUBAL3 | 0.872303 | -2.04716 | 0.040642 | 0.765354 | 0.994198 |
| 71 | PINK1 | 1.266413 | 2.039421 | 0.041408 | 1.009244 | 1.58911 |
| 72 | AR | 1.278172 | 2.016175 | 0.043782 | 1.006866 | 1.622582 |
| 73 | KIF2C | 0.863548 | -1.99112 | 0.046467 | 0.747429 | 0.997707 |
| 74 | CTSG | 1.106146 | 1.973346 | 0.048456 | 1.000684 | 1.222722 |

Supplementary Table 6. Univariate Cox regression analysis of PCD-related gene in GSE84433 cohort.

|  | gene | HR | z | pvalue | lower | upper |
| --- | --- | --- | --- | --- | --- | --- |
| 1 | TUBB6 | 1.37272 | 4.03964 | 5.35E-05 | 1.177144 | 1.60079 |
| 2 | IFNG | 0.711838 | -4.00028 | 6.33E-05 | 0.602634 | 0.840829 |
| 3 | RHOB | 1.304487 | 3.762094 | 0.000168 | 1.135791 | 1.498239 |
| 4 | PDK4 | 1.199135 | 3.648234 | 0.000264 | 1.08767 | 1.322023 |
| 5 | AKR1C2 | 1.168018 | 3.608893 | 0.000308 | 1.07354 | 1.27081 |
| 6 | TUBB2A | 1.352835 | 3.513684 | 0.000442 | 1.142971 | 1.601232 |
| 7 | TPD52L1 | 1.262613 | 3.458401 | 0.000543 | 1.106312 | 1.440995 |
| 8 | DYNC1I1 | 1.255966 | 3.361583 | 0.000775 | 1.099687 | 1.434454 |
| 9 | HSPB8 | 1.162868 | 3.30821 | 0.000939 | 1.063424 | 1.271611 |
| 10 | GBP5 | 0.822512 | -3.27525 | 0.001056 | 0.731748 | 0.924533 |
| 11 | GSN | 1.2947 | 3.226187 | 0.001255 | 1.106686 | 1.514656 |
| 12 | TUBB3 | 1.298248 | 3.122969 | 0.00179 | 1.102084 | 1.529328 |
| 13 | CDO1 | 1.289631 | 3.061585 | 0.002202 | 1.095842 | 1.51769 |
| 14 | STAT1 | 0.773759 | -3.03463 | 0.002408 | 0.655632 | 0.91317 |
| 15 | CAV1 | 1.200016 | 2.96226 | 0.003054 | 1.063637 | 1.353882 |
| 16 | SYNPO2 | 1.126431 | 2.914682 | 0.003561 | 1.039767 | 1.220319 |
| 17 | CTSF | 1.237742 | 2.909538 | 0.00362 | 1.07209 | 1.428988 |
| 18 | COX7A1 | 1.213801 | 2.906395 | 0.003656 | 1.065127 | 1.383227 |
| 19 | BGN | 1.232921 | 2.897006 | 0.003767 | 1.070073 | 1.420554 |
| 20 | VLDLR | 1.330785 | 2.871963 | 0.004079 | 1.094992 | 1.617355 |
| 21 | PLIN4 | 1.124588 | 2.86842 | 0.004125 | 1.037887 | 1.218532 |
| 22 | ADGRE2 | 0.754879 | -2.8025 | 0.005071 | 0.620111 | 0.918937 |
| 23 | EMP1 | 1.226384 | 2.682141 | 0.007315 | 1.056484 | 1.423606 |
| 24 | SOX15 | 1.194044 | 2.6802 | 0.007358 | 1.048811 | 1.359389 |
| 25 | NOX4 | 1.27294 | 2.656815 | 0.007888 | 1.065344 | 1.52099 |
| 26 | MID2 | 1.288665 | 2.646088 | 0.008143 | 1.06797 | 1.554966 |
| 27 | PDGFRB | 1.218591 | 2.630702 | 0.008521 | 1.051697 | 1.411968 |
| 28 | RBM24 | 1.299641 | 2.60148 | 0.009282 | 1.066764 | 1.583356 |
| 29 | FNDC5 | 1.238413 | 2.589308 | 0.009617 | 1.053347 | 1.455994 |
| 30 | EML1 | 1.193248 | 2.551347 | 0.010731 | 1.041803 | 1.366709 |
| 31 | CDC25A | 0.788841 | -2.54131 | 0.011044 | 0.656967 | 0.947187 |
| 32 | IGFBP6 | 1.17813 | 2.500788 | 0.012392 | 1.036087 | 1.339645 |
| 33 | HELLS | 0.780159 | -2.45963 | 0.013908 | 0.640132 | 0.950818 |
| 34 | CHEK2 | 0.75062 | -2.3632 | 0.018118 | 0.591694 | 0.952232 |
| 35 | SRPX | 1.130006 | 2.363119 | 0.018122 | 1.02107 | 1.250563 |
| 36 | EZH2 | 0.793917 | -2.34952 | 0.018798 | 0.654889 | 0.96246 |
| 37 | TMEM238L | 0.850123 | -2.3461 | 0.018971 | 0.742283 | 0.973629 |
| 38 | RIPK2 | 0.78525 | -2.33244 | 0.019678 | 0.640889 | 0.96213 |
| 39 | CPEB1 | 1.503404 | 2.320215 | 0.020329 | 1.065354 | 2.12157 |
| 40 | SLC25A4 | 1.198535 | 2.300098 | 0.021443 | 1.027142 | 1.398526 |
| 41 | IL13RA2 | 1.195575 | 2.279179 | 0.022656 | 1.025334 | 1.394083 |
| 42 | BRCA1 | 0.783183 | -2.27733 | 0.022767 | 0.634626 | 0.966516 |
| 43 | MYB | 0.878904 | -2.25311 | 0.024252 | 0.785555 | 0.983346 |
| 44 | PARP14 | 0.799675 | -2.2043 | 0.027504 | 0.655524 | 0.975525 |
| 45 | SCG2 | 1.138984 | 2.203379 | 0.027568 | 1.014481 | 1.278768 |
| 46 | PINK1 | 1.30376 | 2.193327 | 0.028284 | 1.028624 | 1.652488 |
| 47 | GADD45B | 1.216418 | 2.109187 | 0.034928 | 1.013957 | 1.459305 |
| 48 | LAMP3 | 0.859317 | -2.07673 | 0.037826 | 0.744748 | 0.991511 |
| 49 | RRM2 | 0.860123 | -2.06275 | 0.039137 | 0.745388 | 0.99252 |
| 50 | MAP6 | 1.228238 | 2.059049 | 0.03949 | 1.009942 | 1.493718 |
| 51 | CYSLTR1 | 0.794091 | -2.02088 | 0.043292 | 0.634978 | 0.993074 |
| 52 | CDC25B | 0.843073 | -2.0154 | 0.043863 | 0.714117 | 0.995316 |
| 53 | DUSP1 | 1.162346 | 2.013766 | 0.044034 | 1.004027 | 1.34563 |
| 54 | TNFRSF10B | 0.814975 | -2.00633 | 0.044821 | 0.667333 | 0.995283 |
| 55 | RUFY4 | 0.642446 | -2.00625 | 0.044829 | 0.416973 | 0.989843 |
| 56 | TMEM74 | 1.616355 | 1.993579 | 0.046198 | 1.008129 | 2.591537 |
| 57 | TRIM59 | 0.702174 | -1.99337 | 0.046221 | 0.495979 | 0.994092 |
| 58 | TUBAL3 | 0.865203 | -1.98181 | 0.047501 | 0.749771 | 0.998405 |
| 59 | CGAS | 0.819097 | -1.9603 | 0.049961 | 0.670943 | 0.999966 |

Supplementary Table 7. Comparation of Top20 genes mutation frequency between low PCDS group and high PCDS group.

|  | Hugo_Symbol | High | Low | pval | or | ci.up | ci.low |
| --- | --- | --- | --- | --- | --- | --- | --- |
| 1 | **FAT4** | 25 | 56 | 0.000155 | 0.368926 | 0.637618 | 0.208784 |
| 2 | **LRP1B** | 40 | 70 | 0.001026 | 0.465701 | 0.750648 | 0.286151 |
| 3 | **FAT3** | 21 | 44 | 0.002574 | 0.418908 | 0.757744 | 0.225587 |
| 4 | **SYNE1** | 38 | 64 | 0.003782 | 0.500908 | 0.81564 | 0.304649 |
| 5 | **TTN** | 88 | 118 | 0.003993 | 0.541921 | 0.829595 | 0.352663 |
| 6 | **PCLO** | 26 | 49 | 0.004441 | 0.463595 | 0.805138 | 0.262125 |
| 7 | **ARID1A** | 36 | 61 | 0.004711 | 0.502927 | 0.826053 | 0.302999 |
| 8 | **KMT2D** | 21 | 42 | 0.00557 | 0.444718 | 0.807979 | 0.23868 |
| 9 | **OBSCN** | 25 | 47 | 0.005811 | 0.468363 | 0.820755 | 0.262223 |
| 10 | **ZFHX4** | 23 | 43 | 0.009836 | 0.478145 | 0.854887 | 0.261851 |
| 11 | **HMCN1** | 26 | 45 | 0.017622 | 0.518769 | 0.9078 | 0.291595 |
| 12 | **CSMD1** | 31 | 48 | 0.043101 | 0.585736 | 0.996656 | 0.340403 |
| 13 | MUC16 | 54 | 73 | 0.050977 | 0.648015 | 1.016487 | 0.411378 |
| 14 | FLG | 38 | 53 | 0.092876 | 0.656396 | 1.083299 | 0.394934 |
| 15 | SPTA1 | 29 | 41 | 0.145845 | 0.664104 | 1.15671 | 0.377401 |
| 16 | RYR2 | 30 | 38 | 0.34973 | 0.760419 | 1.330302 | 0.431582 |
| 17 | DNAH5 | 31 | 38 | 0.42557 | 0.790626 | 1.377921 | 0.450939 |
| 18 | CSMD3 | 44 | 50 | 0.553369 | 0.856241 | 1.401197 | 0.521957 |
| 19 | PCDH15 | 30 | 35 | 0.586651 | 0.841454 | 1.486741 | 0.473927 |
| 20 | TP53 | 92 | 97 | 0.683877 | 0.919622 | 1.400984 | 0.603348 |

Supplementary Table 8. The comparation of C-index between nomogram and TNM stage in TCGA, GSE84437 and GSE84433 cohorts.

| Model | C-index | 95%CI | P value* |
| --- | --- | --- | --- |
| TCGA cohort |  |  |  |
| Nomogram | 0.653 | (0.608, 0.698) | < 0.001 |
| TNM | 0.574 | (0.525, 0.623) |  |
| GSE84437 cohort |  |  |  |
| Nomogram | 0.657 | (0.622, 0.692) | <0.001 |
| TNM | 0.597 | (0.572, 0.622) |  |
| GSE84433 cohort |  |  |  |
| Nomogram | 0.654 | (0.614, 0.693) | <0.001 |
| TNM | 0.598 | (0.571, 0.625) |  |

* DeLong’s test
