# Supplementary material for: Multiple programmed cell death patterns predict the prognosis and drug sensitivity in gastric cancer
Source: Front Immunol. 2025 Feb 4;16:1511453. doi: 10.3389/fimmu.2025.1511453 (PMC11832517; doi:10.3389/fimmu.2025.1511453)
Supplement: Supplementary file 4 [file Table4.docx]

Supplementary Material

## Supplementary Figures

##
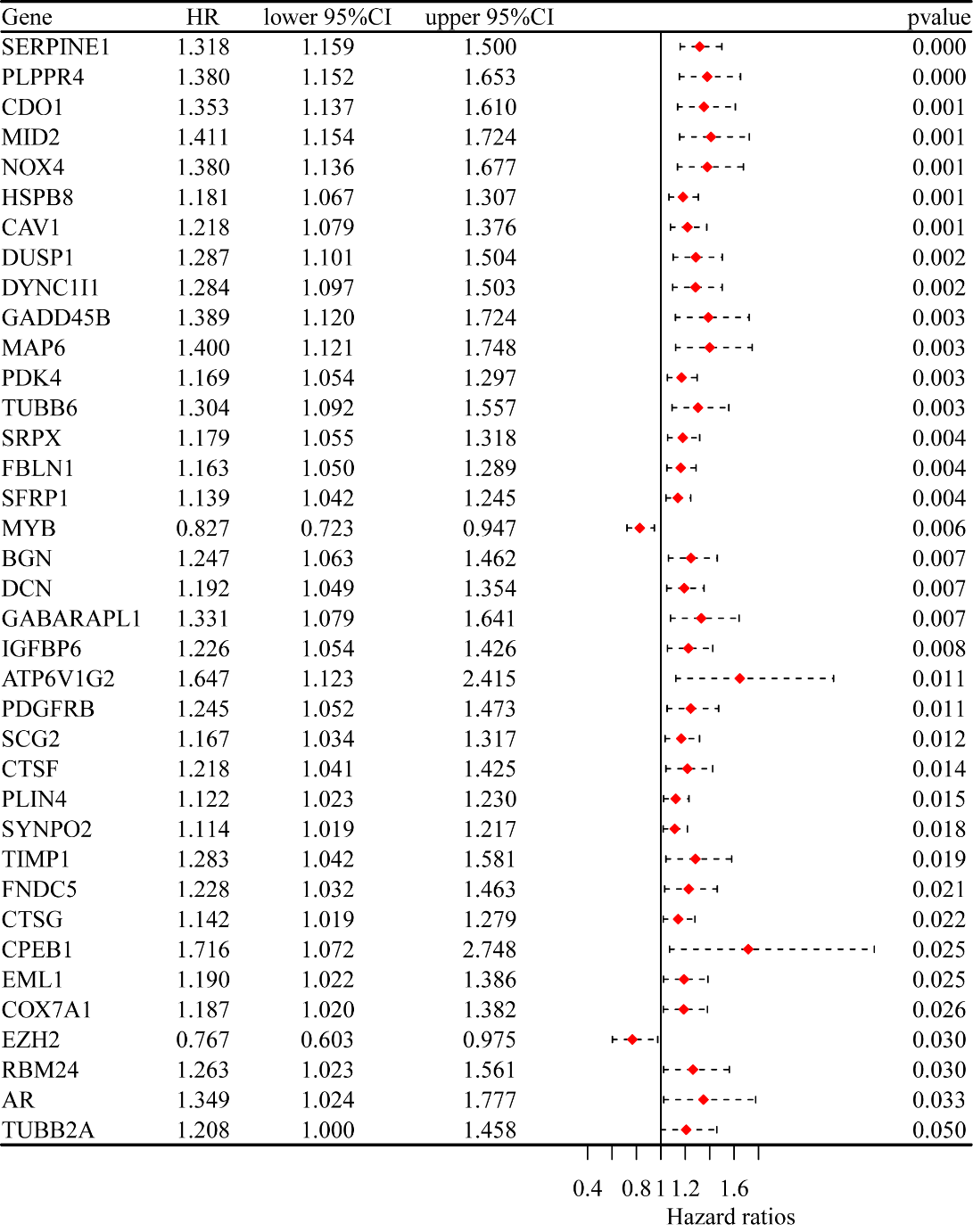


**Supplementary Figure 1.** Forest plot for intersection results of univariate cox regression analysis of the TCGA and GSE84437 cohorts.


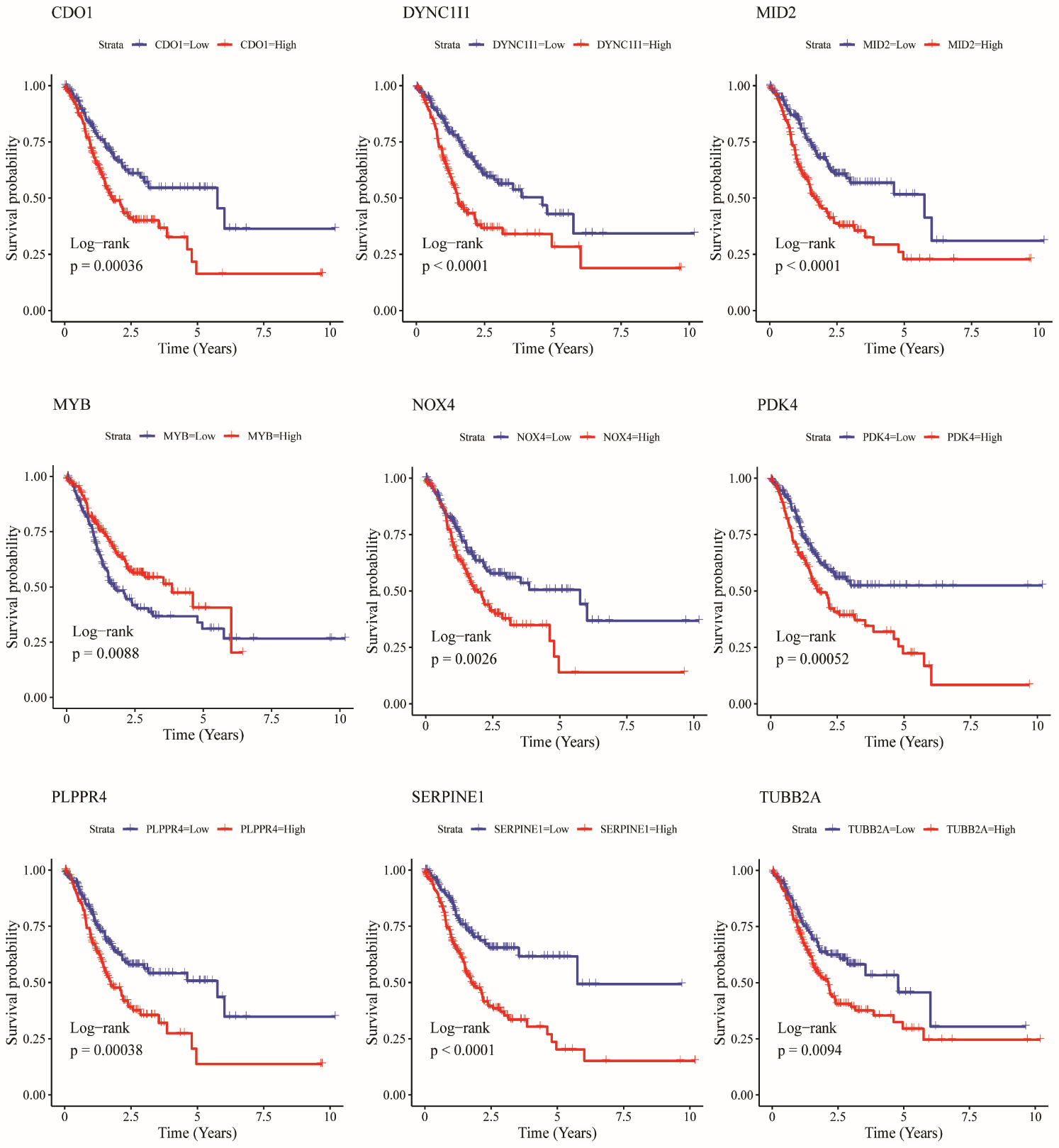


**Supplementary Figure 2.** Kaplan-Meier analysis of each model gene**.**

**
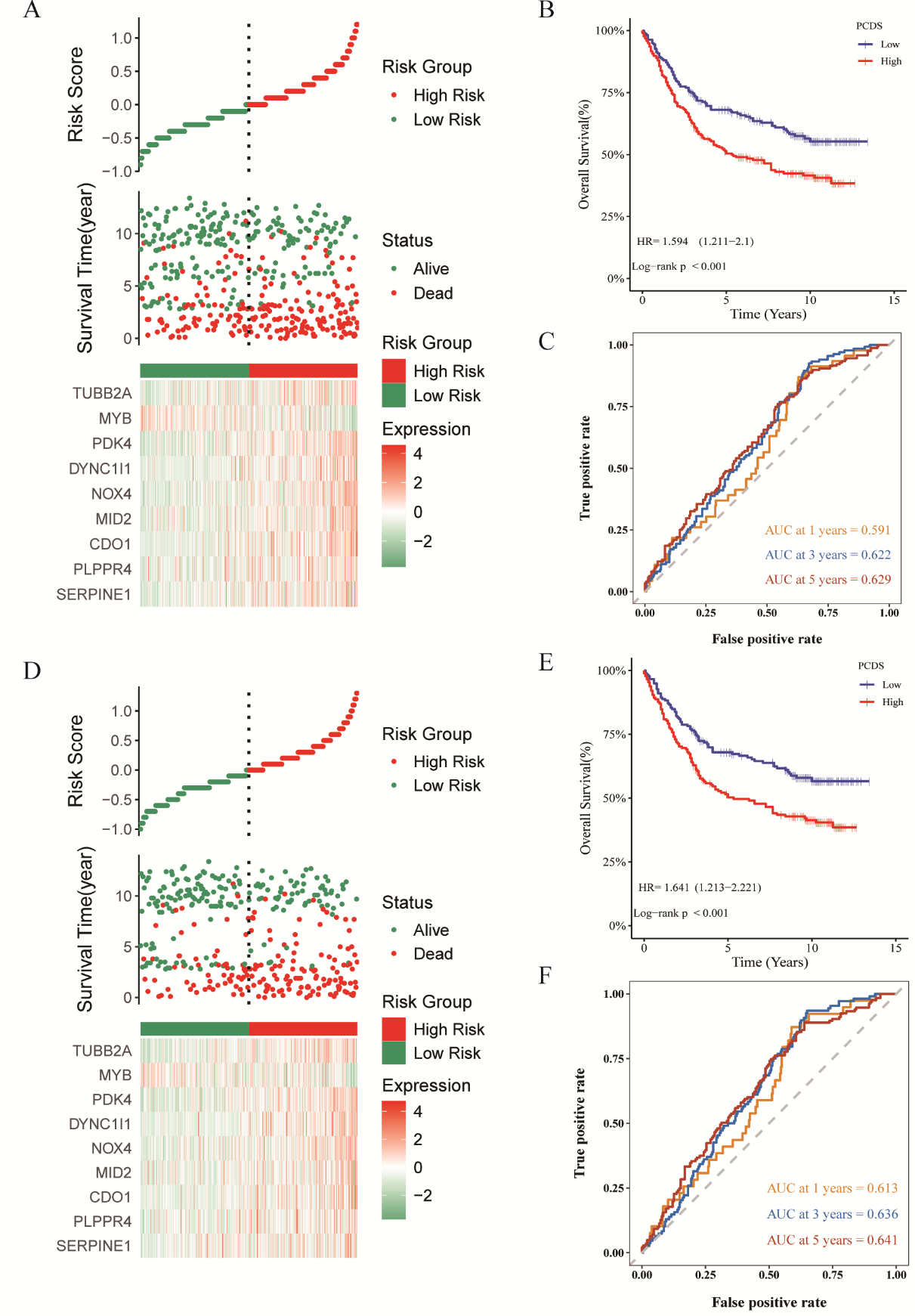
**

**Supplementary Figure 3.** External validation of the PCDS predicting value. (**A, D**) Distribution of PCDS, survival status and time, heatmap of PCD signature including 9 genes in GSE84437 and GSE 84433 cohorts. (**B, E**) KM curves of PCDS predicting the OS of patients in GSE84437 and GSE 84433 cohorts. (**C, F**) Time dependent ROC analysis of the PCDS predicting the OS of patients in GSE84437 and GSE 84433 cohorts.

**
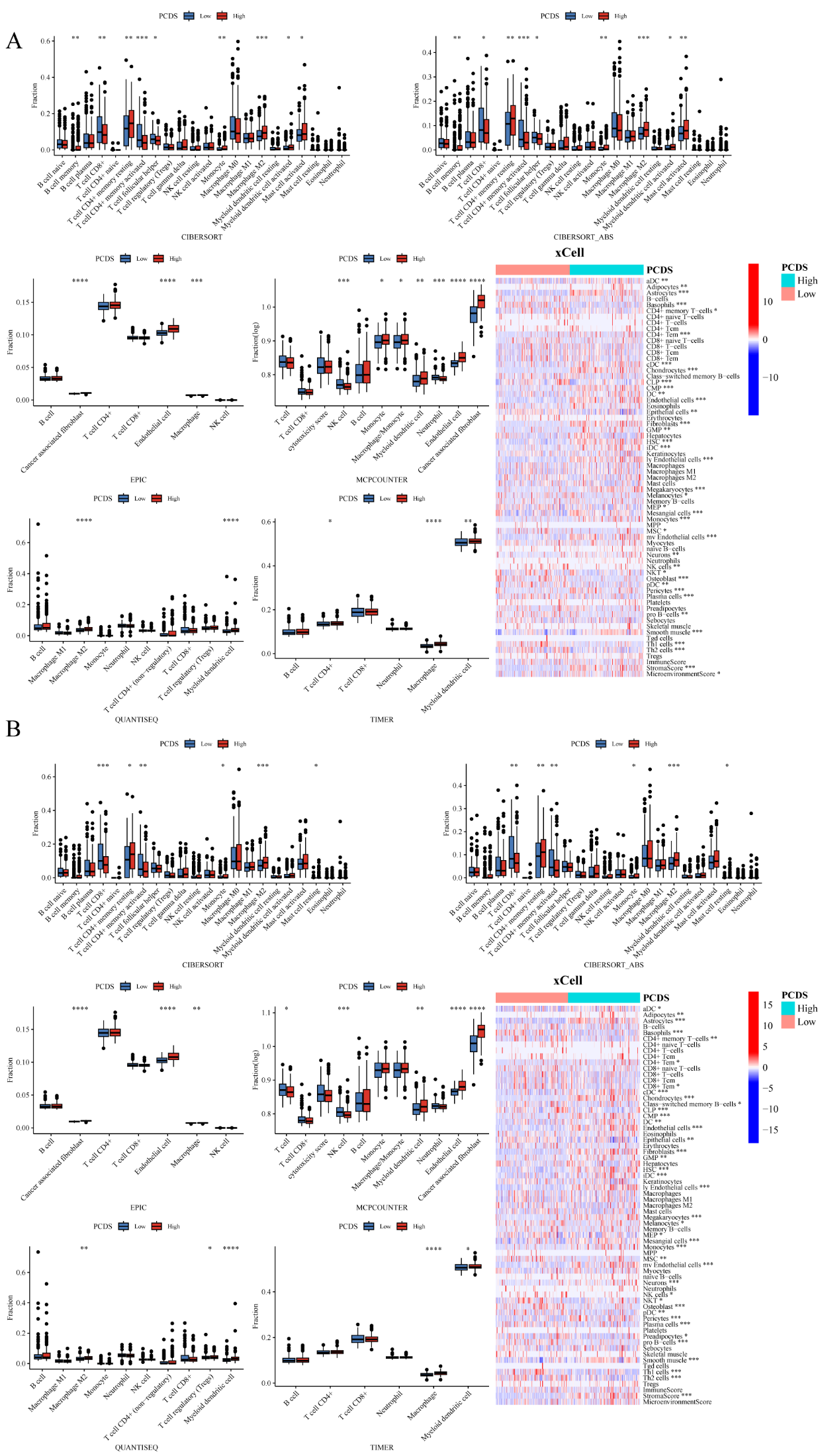
**

**Supplementary Figure 4.** Dissection of TIME based on PCDS signature. (**A**) In GSE 84437 cohort. Bar plot of the correlation between immunomodulators and the PCDS in GC patients; Association between PCDS and tumor microenvironment by Estimate algorithms; Association between PCDS and stromal and immune infiltration estimations by TIMER, CIBERSORT, quanTIseq, xCell, MCP-counter and EPIC algorithms. (**B**) In GSE 84433 cohort. Bar plot of the correlation between immunomodulators and the PCDS in GC patients; Association between PCDS and tumor microenvironment by Estimate algorithms; Association between PCDS and stromal and immune infiltration estimations by TIMER, CIBERSORT, quanTIseq, xCell, MCP-counter and EPIC algorithms. *** means P < 0.001; ** means P < 0.01; * means P < 0.05.


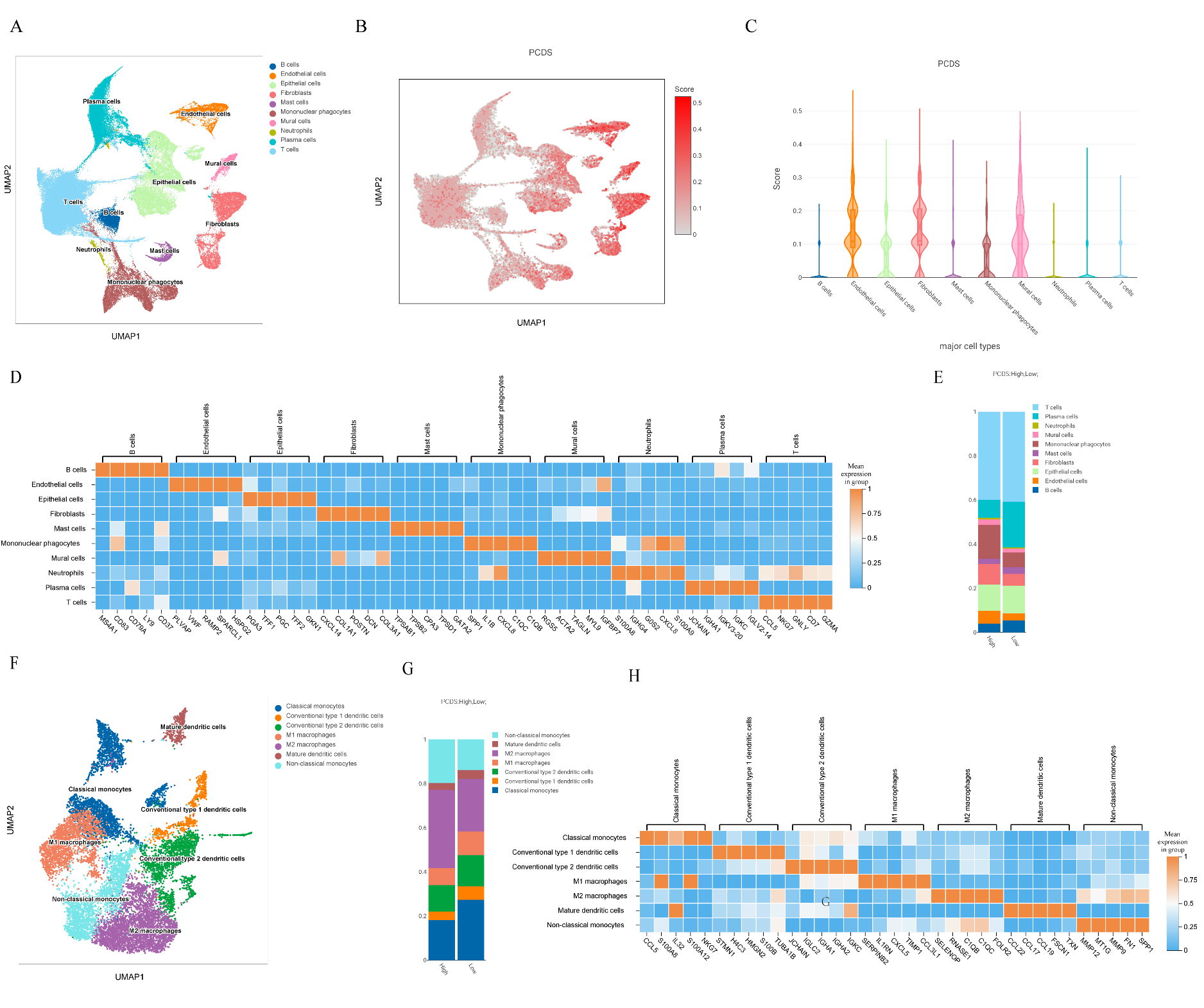


**Supplementary Figure 5.** Single cell RNA sequence (scRNA-seq) profiling for PCDS related genes. (**A**) The UMAP plot provides an annotation and color codes for the different cell types present in GC patients. (**B-C**) The distribution of PCDS in each cell types. (**D**)  Marker genes in each cell types. (**E**) Histograms shows the percentage of cell types between high and low PCDS groups. (**F**) The UMAP plot shows the clustering of mononuclear phagocytes. (**G**) Histograms shows the proportion of different mononuclear phagocyte clusters in the high PCDS and low PCDS groups. (**H**)Marker genes for the clustering of mononuclear phagocytes.

**
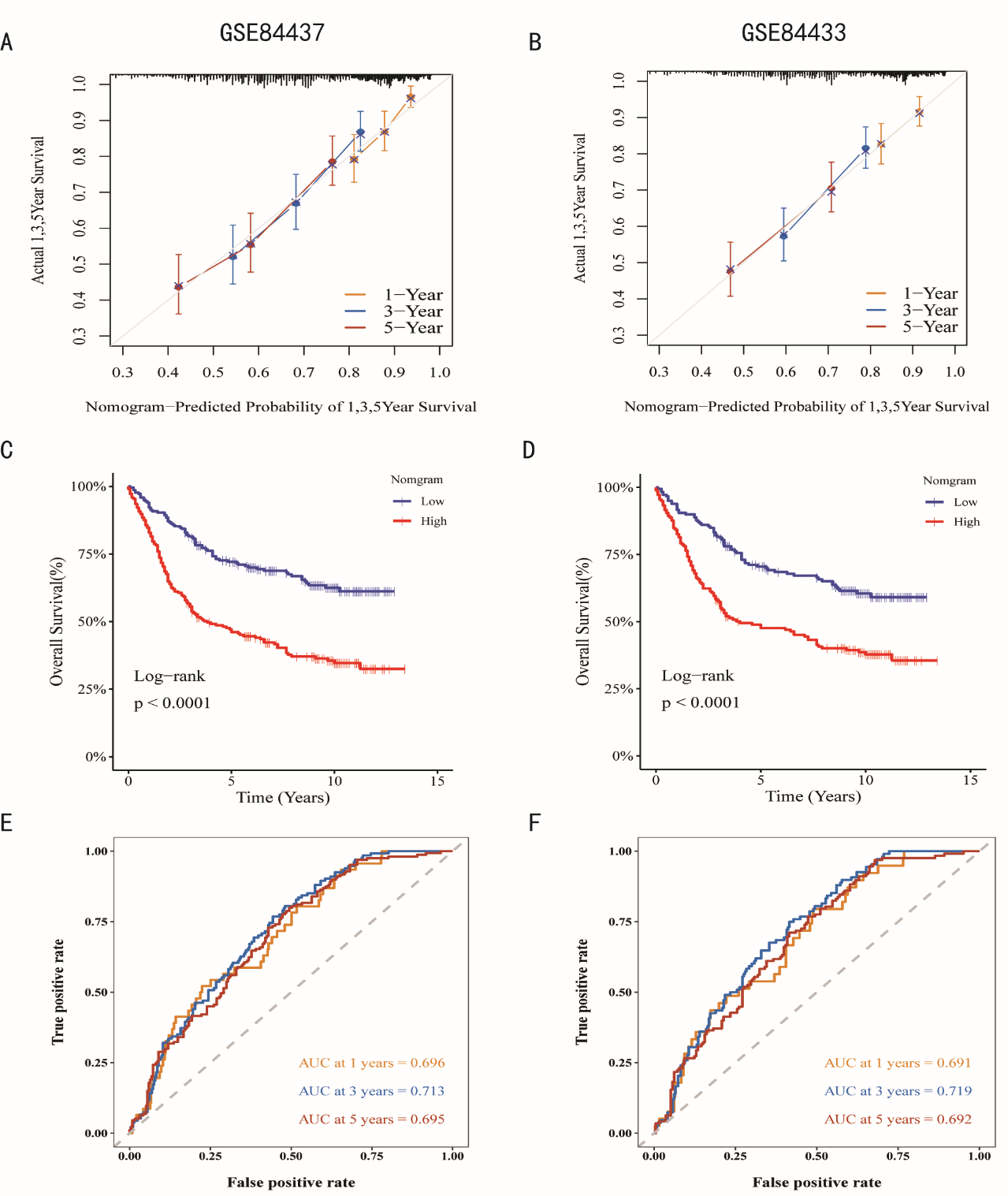
**

**Supplementary Figure 6.** External validation of the nomogram survival model. (**A, B**) The calibration curve of the nomogram in GSE84437 and GSE84433 cohort. (**C, D**) Kaplan-Meier analyses based on the nomogram score in GSE84437 and GSE84433 cohort. **(E, F)** Receiver operator characteristic (ROC) analysis of nomogram in GSE84437 and GSE84433 cohort.


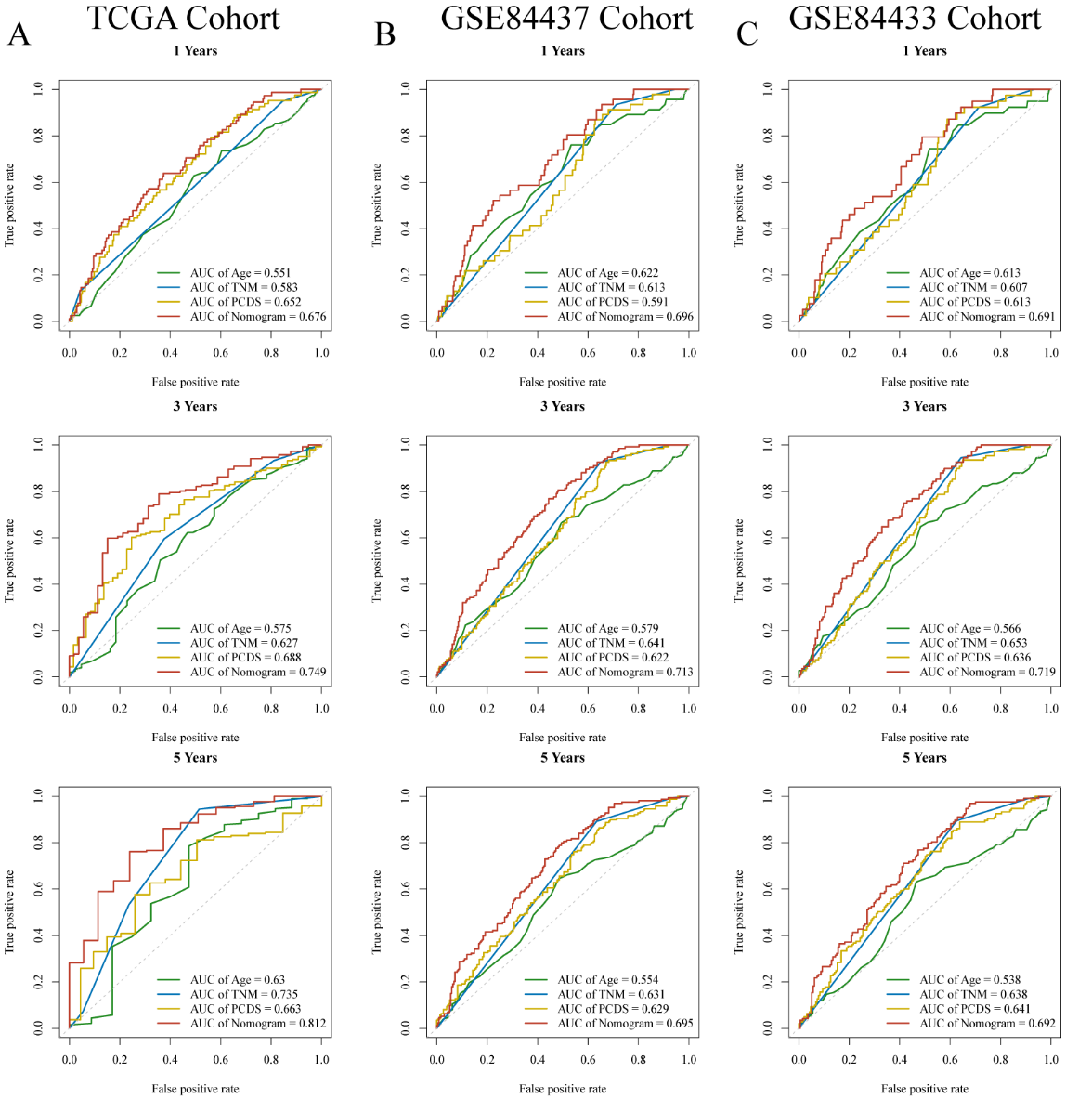


**Supplementary Figure 7** Time dependent ROC curves for different models in GC patients. (**A**) Time dependent ROC curves at 1, 3, 5 years for different models in GC patients in TCGA cohort. (**B**) Time dependent ROC curves at 1, 3, 5 years for different models in GC patients in GSE84437cohort. (**C**) Time dependent ROC curves at 1, 3, 5 years for different models in GC patients in GSE84433 cohort.


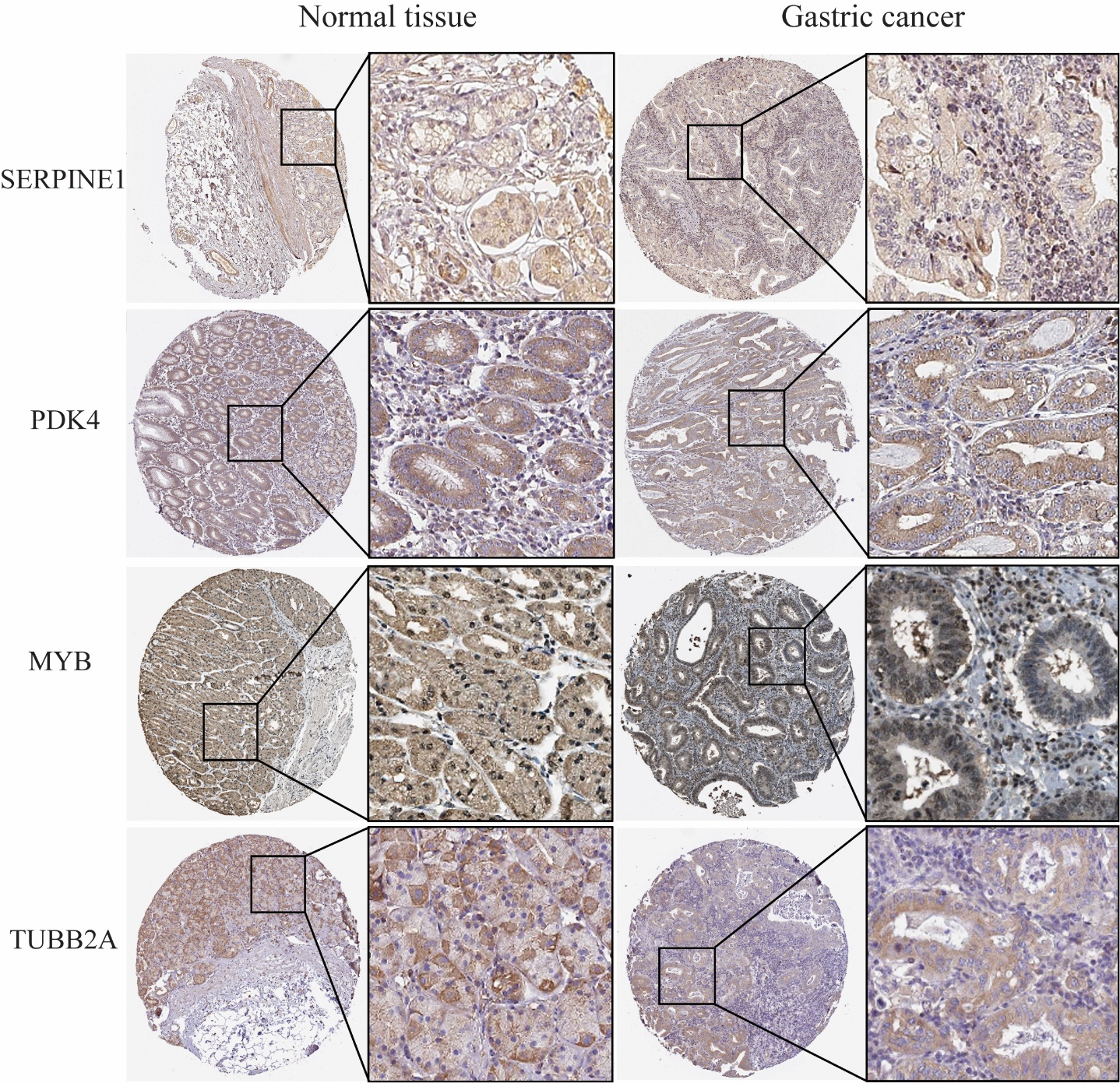


**Supplementary Figure 8.** The protein expression levels of SERPINE1, PDK4, MYB and TUBB2A in gastric normal tissues and gastric cancers from HPA online database.

**
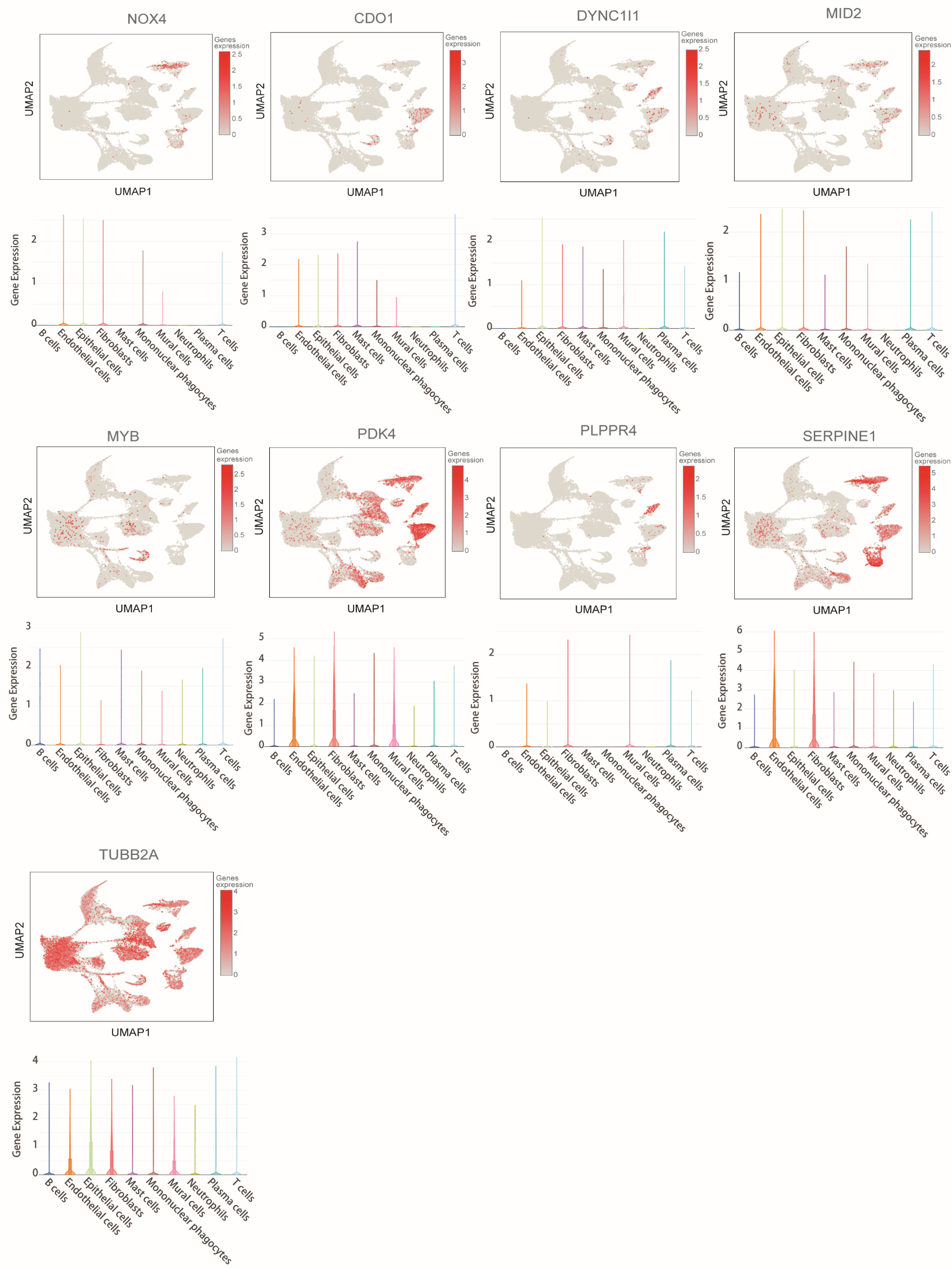
****Supplementary Figure 9.** The expression of genes in the PCDS related gene in each cell type.
